# Genomic Evidence for the Purging of Deleterious Genetic Variation in the Endangered North Atlantic Right Whale

**DOI:** 10.1111/eva.70055

**Published:** 2024-12-23

**Authors:** Richard W. Orton, Philip K. Hamilton, Timothy R. Frasier

**Affiliations:** ^1^ Department of Biology Saint Mary's University Halifax Nova Scotia Canada; ^2^ Anderson Cabot Center for Ocean Life New England Aquarium Boston Massachusetts USA

**Keywords:** genetic purging, inbreeding, mutation load, North Atlantic right whale

## Abstract

The reduced genetic diversity and frequent inbreeding associated with small population size may underpin the accumulation and expression of deleterious mutations (mutation load) in some declining populations. However, demographic perturbations and inbreeding coupled with purifying selection can also purge declining populations of deleterious mutations, leading to intriguing recoveries. To better understand the links between deleterious genetic variation and population status, we assess patterns of genetic diversity, inbreeding, and mutation load across the genomes of three species of *Balaenidae* whale with different demographic histories and recoveries following the end of commercial whaling in the 1980s. Unlike bowhead (BH) and Southern right whales (SRW), which show signs of recent recovery, reproductive rates of the endangered North Atlantic right whale (NARW) remain lower than expected. We show that the NARW is currently marked by low genetic diversity, historical inbreeding, and a high mutation load. Still, we reveal evidence that genetic purging has reduced the frequency of highly deleterious alleles in NARW, which could increase chances of future population recovery. We also identify a suite of mutations putatively linked to congenital defects that occur at high frequencies in nulliparous NARW females but are rare in NARW with high reproductive success. These same mutations are nearly absent in BH and SRW in this study, suggesting that the purging of key variants may shape the probability of population recovery. As anthropogenic disturbances continue to reduce the sizes of many populations in nature, resolving the links between population dynamics and mutation load could become increasingly important.

## 1 Introduction

The effects of deleterious genetic variation segregating within large and stable populations (of diploid organisms) can be difficult to quantify because of high levels of heterozygosity. Specifically, deleterious genetic variation is often at least partially recessive (Yang et al. 2017), and is therefore less likely to observably affect the reproductive success (RS) of individuals when sampled from heterozygous genotypes (Morton, Crow, and Muller 1956). Conversely, the accumulation of deleterious genetic variation, termed “mutation load” (Muller 1950), can critically impact declining populations due to the increased rates of inbreeding and genetic drift that are inherent consequences of smaller population size (Frankham 1998). Inbreeding, in particular, reduces genome‐wide levels of heterozygosity. Heterozygous genotypes that mask the effects of recessive deleterious alleles (masked mutation load) can then be replaced with homozygous genotypes from which deleterious alleles are inevitably expressed (realized mutation load) (Bertorelle et al. 2022). Because the replacement of the masked mutation load with the realized mutation load is the root of inbreeding depression (Charlesworth and Charlesworth 1987), characterizing the processes that shape the distribution of mutation load across genomes could inform efforts to mitigate inbreeding depression in declining populations (Teixeira and Huber 2021; Humble et al. 2023).

The distribution of mutation load across genomes is shaped by complex processes that involve different mechanisms of evolution (Haldane 1937; Agrawal and Whitlock 2012). Genetic purging, for example, reduces the frequency of deleterious genetic variation in populations through demographic processes (e.g., population contractions) as well as through purifying selection that targets recessive alleles harbored in homozygous stretches of genomes (Barrett and Charlesworth 1991; Dussex et al. 2023). Genetic purging through inbreeding, in particular, may be relevant to conservation efforts because inbreeding depression is a common cause of population decline (Pérez‐Pereira, Caballero, and García‐Dorado 2022). Although few studies have directly connected genetic purging to inbreeding (but see Khan et al. 2021), a wide breadth of taxa have been represented across studies inferring broad signatures of genetic purging (e.g., mountain gorillas [ *Gorilla gorilla* ] (Xue et al. 2015); rattlesnakes [ *Sistrurus catenatus* ] (Ochoa and Gibbs 2021); and Alpine ibex [ *Capra ibex* ] (Grossen et al. 2020)), suggesting that genetic purging could be common in nature. However, many of these studies regarded only genome‐wide differences in mutation load or differences in deleterious allele frequencies between populations with contrasting demographic histories, leaving gaps in our understanding of genetic purging. To better characterize genetic purging and to ascertain the extent to which it occurs through inbreeding in nature, studies may need to more thoroughly explore the relationships between deleterious genetic variation and inbreeding (Agrawal and Whitlock 2012; Dussex et al. 2023).

One potentially important but often overlooked characteristic of genetic purging is that its efficacy may vary across genomic regions (Kleinman‐Ruiz et al. 2022; Smeds and Ellegren 2023). Chiefly, deleterious alleles cannot be masked from selection on sex chromosomes in the heterogametic sex of species with hemizygous sex chromosomes (Haldane 1922). In turn, hemizygosity can influence the efficacy of genetic purging via either one of two different paths. First, ongoing “exposure” of deleterious alleles to selection in the heterogametic sex could maintain deleterious alleles at low frequencies and reduce the number of sites segregating deleterious genetic variation altogether. With a masked mutation load approaching zero, inbreeding or demographic stochasticity would not increase the realized mutation load to appreciable levels, and genetic purging would leave only a superficial footprint across sex chromosomes (as in Kleinman‐Ruiz et al. 2022). Second, segregating deleterious alleles could rise to higher frequencies more quickly on sex chromosomes due to accelerated genetic drift (Chen et al. 2018). Under this scenario, inbreeding and demographic stochasticity could quickly increase the realized mutation load considerably. The abrupt exposure of deleterious alleles to selection through hemizygosity could then leave a quantifiable footprint of genetic purging across sex chromosomes (as in Smeds and Ellegren 2023). Individuals of the homogametic sex of large populations would perceivably shelter unexpectedly high masked mutation loads given the latter. Therefore, resolving the relationships between hemizygosity and genetic purging could inform conservation decisions pertaining to sex ratios and the genetic rescue of small and declining populations.

The population sizes of many baleen whales were greatly reduced by commercial whaling that began as early as 1100 AD and continued until an international whaling moratorium in the mid‐1980s (Clapham and Baker 2018). Although many populations show current signs of recovery (Clapham and Baker 2018), there are exceptions, such as the critically endangered North Atlantic right whale ( *Eubalaena glacialis* ) [NARW]. Current estimates predict that little more than 350 NARW remain, and since monitoring began in 1980, the census size of NARW has not exceeded 500 (Pettis, Pace III, and Hamilton 2021). This contrasts with the histories of other *Balaenidae* whales, including Southern right whales ( *Eubalaena australis* [SRW]) in the Southwest Atlantic and Bowhead whales from the Eastern Svalbard Barents Sea ( *Balaena mysticetus* [BH]), which despite intensive whaling, show signs of population recovery (Arias et al. 2018; Cerca et al. 2022). Both SRW and BH have sustained larger population sizes than NARW throughout their histories, which could have underpinned reduced levels of inbreeding and genetic drift, and thus reduced mutation load (Cerca et al. 2022; Crossman, Fontaine, and Frasier 2023). In light of overall poorer health and reduced calving rates (Pettis, Pace III, and Hamilton 2021), both consequences of inbreeding depression (Charlesworth and Willis 2009), quantifying and characterizing mutation load in NARW may provide insight into one of the mechanisms potentially underlying the species' lack of population recovery.

Here, we used whole genome data to characterize genetic purging across *Balaenidae* and gauged the potential impact of deleterious genetic variation on the absence of population recovery of NARW. First, we quantified variation in genetic diversity and mutation load across NARW, SRW, and BH. We then leveraged variation in deleterious allele and genotype frequencies against inbreeding statistics to test relationships between mutation load and inbreeding. These relationships were explored independently for the autosomes and X chromosome to understand how genetic purging may vary across genomic regions. We also considered the different time frames of inbreeding to more thoroughly understand its impact on mutation load. Last, we assessed site‐level differences in genetic diversity and mutation load across 10 NARW females with different reproductive histories. To identify and characterize putative variants associated with differences in RS in NARW females, we coupled three outlier approaches with two gene enrichment tests. The results of this study will inform conservation strategies for NARW and could improve our knowledge of mutation load in imperiled populations.

## 2 Materials and Methods

### 2.1 Sample Selection and Sequencing

In Crossman, Fontaine, and Frasier (2023), 12 NARW and 10 SRW samples were selected for whole genome sequencing at a targeted coverage of 40×. DNA was stored at −20°C at Saint Mary's University and extracted using a standard phenol–chloroform–isoamyl protocol. The authors selected five nulliparous female NARW and five NARW females with the highest number of calves observed since monitoring began in 1980 (Table S1). The authors also selected two male NARW, five male SRW, and five female SRW. NARW samples were selected from across the species' range and SRW genomes were sampled from winter calving grounds in Peninsula Valdes, Argentina (*n* = 9) and summer feeding grounds off the coast of South Georgia (*n* = 1). Note that both microsatellite loci and mitochondrial gene fragment analyses show that SRW sampled from South Georgia during the summer are genetically indistinguishable from SRW from Argentina (Carroll et al. 2020). In addition, whole genome analyses show no population stratification across NARW (Crossman, Fontaine, and Frasier 2023). For each chosen sample, Crossman, Fontaine, and Frasier (2023) sent 1‐5ug of genomic DNA to the McGill Applied Genomics Innovation Core (Montreal, Quebec, Canada) where samples were PCR‐free library prepped using a NxSeq AmpFREE kit (Lucigen, Wisconsin, USA) and pooled across three Illumina NovaSeq 6000 S4 lanes using 150‐bp paired‐end reads. Here, we used these sequences to address questions regarding mutation load. To extend the scope of this study, we also included Short Read Archive data with an average coverage of 20× from 12 BH whales (four females and eight males). These genomes were sampled from the Eastern Svalbard Barents Sea during a different population genetics study (Cerca et al. 2022). These data are publicly available through the National Center for Biotechnology Information and were downloaded as unfiltered fastq files. A summary of all samples with population genomic summary statistics is shown in Table S2.

### 2.2 Processing Reads and Variant Calling

Raw right whale reads were demultiplexed and adapter trimmed at the core facility. We further trimmed and filtered raw reads using thresholds implemented with Trimmomatic v0.39 (Bolger, Lohse, and Usadel 2014). We removed reads with Phred scores below 30, a minimum length of 32, average quality below 32, or leading or trailing ends with scores below 20. Reads that passed thresholds were mapped to the first 22 scaffolds of the blue whale (*Baelenoptera musculus*) reference genome (Bukhman et al. 2022), representing its 21 autosomes and the X chromosome. We chose to use the m3BalMus1.pri.V3 assembly as our reference because the assembly is high quality (2.4 Gb comprised of 972 contigs with scaffoldN50 of 110.3 Mb) and mapping reads to an outgroup helps to avoid ascertainment bias when assessing mutation load (Smeds and Ellegren 2023). Reads were sorted, merged, and indexed using SAMtools v1.17 (Danecek et al. 2021). We used BWA v0.7.12 mem to map reads (Li and Durbin 2009) and GATK v4.1.0 (McKenna et al. 2010) to call variants. We followed the Broad Institute's best practices workflow that included marking duplicates with Picard v1.54 and calling population‐level variation using GATK's HaplotypeCaller and GenotypGVCF modules. We did not remove invariant sites as to accurately estimate population genomic summary statistics. We excluded repetitive regions using VCFtools v0.1.13 (Danecek et al. 2011) *exclude*‐*bed* and the blue whale repeat masker bed file (Bukhman et al. 2022). In addition to our initial filtering steps that followed the Broad Institute's best practices guidelines, we filtered indels and genotype calls below a quality of 30. We also filtered calls that were either below a read depth of 15 or above twice the average read depth, which could represent duplicated regions. We normalized multiallelic sites using BCFtools v1.11 *normalize* ‐m command (Danecek et al. 2021). For downstream analyses, we parsed the autosomes and X chromosome and removed male samples from our analyses of the X chromosome using VCFtools, as males will be “homozygous” for that scaffold. To avoid spurious results caused by missing data, we used VCFtools to remove any sites that had even a single missing genotype call. This resulted in two gVCF files with zero missing data and 43,205,835 total sites retained across autosomes and 4,004,569 total sites retained for the X chromosome.

### 2.3 Phylogenetic Visualization

To estimate and visualize phylogenetic relationships across *Balaenidae*, we used whole mitochondrial genomes assembled from raw reads using MITOBIM v1.9.1 (Hahn, Bachmann, and Chevreux 2013). This method produced a mitochondrial genome of 16,402 bp with 2081 variant sites. We used this alignment to generate a phylogeny with BEAST v2.6.6 (Drummond and Rambaut 2007). We ran BEAST with a chain length of 10 million MCMC iterations, a burn‐in of 100,000 generations, and a Yule model prior. To avoid overparameterization across our dataset of species with limited genetic diversity, we used a HKY85 (Hasegawa, Kishino, and Yano 1985) substitution model to assess the topology across species. This method is anticipated to be appropriate in cases where there is little information regarding the parameters (Nascimento, Reis, and Yang 2017). The phylogeny was visualized using R package ggtree (Yu 2020) and we labeled nodes according to posterior probability estimates from the maximum clade credibility tree generated using TreeAnnotator (Drummond and Rambaut 2007).

### 2.4 Estimating Genetic Variation and Inbreeding

We used the program PIXY v1.2.7 (Korunes and Samuk 2021) to estimate nucleotide diversity (π) in 100 Kb nonoverlapping windows, and VCFtools *het* to estimate the inbreeding coefficient (*F*) (Hom_observed_—Hom_expected_/N_total_—Hom_expected_) for each individual. We also estimated runs of homozygosity (ROH) for each species using BCFtools v1.11‐*roh*, which utilizes a hidden Markov model to identify stretches of autozygosity (Danecek et al. 2021). In particular, autozygosity was estimated from genotype likelihoods, empirical estimates of derived allele frequencies, and without a genetic map. For ROH analyses, we then used custom scripts to quantify the number of ROH (NROH), the mean length of ROH, the sum of ROH (SROH), and the fraction of the genome found in ROH (FROH) for each individual. For the total length of the genome, we used the sum length of the 21 autosomes and the length of the X chromosome, respectively (Bukhman et al. 2022). Analyses were completed independently for the autosomes and the X chromosome and ROH results were aggregated by species and genomic region to quantify summary statistics for species and genomic region using base R (R Foundation for Statistical Computing 2023).

### 2.5 Quantifying Mutation Load

To identify deleterious genetic variation within and across species, we used SnpEff to annotate variant sites with potential fitness impacts caused by mutation (Cingolani et al. 2012). Notably, this method may have an important advantage over the Genome Evolutionary Rate Profiling (GERP) (Davydov et al. 2010), another common method of annotating predicted fitness impacts of mutation. Recent data show that GERP may be unreliable in mammals due to elevated rates of gene turnover (Huber, Kim, and Lohmueller 2020). SnpEff navigates this issue by creating a novel database using reference genome annotations and expression data (Cingolani et al. 2012). Specifically, SnpEff considers the genomic coordinates and associated nucleotide change of variants to annotate the predicted impact of mutations on protein structure. To annotate the predicted impact of variants, we used information from the blue whale genome assembly that included coding sequences, protein sequences, and mRNA transcripts (Bukhman et al. 2022). In our case, we used the annotations of “modifier,” “low,” “moderate,” and “high” impact, where the modifier category reflects mutations in non‐coding regions of the genome and high impact mutations represent loss of function mutations. The low and moderate impact categories represent synonymous and missense mutations, respectively. Similar to Grossen et al. (2020) and Nigenda‐Morales et al. (2023), we considered an allele “derived” if that allele segregated at the minor allele frequency across *Balaenidae*. Note that Nigenda‐Morales et al. (2023) also used the reference genome of a different species, the minke whale (*Balaenoptera acutorostrata scammony*), to characterize mutation load in fin whales ( *Balaenoptera physalus* ) due to the minke reference genome's high quality assembly. The authors had no reason *post hoc* to believe that the use of this genome impacted their results (Nigenda‐Morales et al. 2023). Following variant annotation and polarization, we assumed derived alleles to be deleterious because deleterious mutations are overwhelmingly derived (Keightley and Lynch 2003). These methods provided annotated gVCF files with 1,426,491 variants across the autosomes and 167,668 variants for the X chromosome.

To quantify mutation load within and across *Balaenidae* species, we summed homozygous and heterozygous genotypes across individual genomes, producing three different estimates of mutation load. First, we estimated the Total mutation load, representing the loss of individual fitness expected to result from the sum of homozygous deleterious genotypes and heterozygous genotypes when the effects of mutation are additive. We then estimated the Homozygous and Heterozygous mutation loads independently, reflecting the respective loss of fitness when deleterious mutations accrue half the impact when sampled from heterozygous sites compared with homozygous sites.

Genotype frequencies were calculated using VCFtools‐hardy, and we weighted mutation impacts predicted by SnpEff: 0, 0.1, 0.3, 0.6 for modifier, low, moderate, and high impact mutations, respectively. Differences in weights reflect previously published selection coefficient distributions (Tamuri, dos Reis, and Goldstein 2012; Kyriazis, Robinson, and Lohmueller 2023). Additionally, weights were assigned so that any individual homozygous for the derived allele at each segregating site in our gVCF would have a total mutation load equal to one. This framework mirrors early equations of fitness loss (e.g., *W* = 1—2*pqhs*—*q* ^2^ *s*) in that a complete loss of fitness theoretically results when an individual is homozygous for the deleterious allele at each coding site (Haldane 1937). We used this method to quantify mutation load for autosomes and the X chromosome independently.

To understand if variation in mutation load is driven by high allele frequencies or differences in the numbers of sites segregating deleterious variation, we assessed mean allele frequencies for each individual and generated unfolded site frequency spectra (SFS) for each species using easySFS (Gutenkunst et al. 2009; Overcast 2023) in Python v3.10.2 (Van Rossum and Drake Jr 1995). Since we made inferences regarding which alleles were ancestral and derived based on phylogenetic information, we used the unfolded SFS, which is based on the minor allele frequency regardless of ancestral state. We also compared the total number of sites that were fixed for the reference allele, fixed for a derived allele, and polymorphic. We preformed this analysis separately for the autosomes and the X chromosome.

### 2.6 Assessing Genetic Purging Through Inbreeding

To assess genetic purging through inbreeding, we first generated estimates of *Rxy* for each species pair for each mutation impact category predicted from SnpEff. This statistic is the ratio of the frequency of a single allele at a single locus in population_x_ compared to the frequency of that same allele at that same locus in population_y_. We used *Rxy* because it is commonly used to infer footprints of genetic purging through its ability to summarize derived allele statistics (Do et al. 2015). Next, we estimated the “relative mutation load” to assess variation in the relationships between homozygous mutation load and inbreeding in each species. In populations where genetic purging through inbreeding has occurred, the homozygous mutation load should be smaller than expected given estimates of inbreeding. This is because purifying selection acts to reduce the frequency of deleterious recessive homozygotes, removing two deleterious alleles from the population per locus (as reviewed by Dussex et al. 2023). We estimated this statistic by dividing homozygous mutation load by *F* for each individual. Last, we tested for the differential enrichment of deleterious homozygous genotypes within ROH. We accomplished this analysis using BEDtools v2.30.0 (Quinlan and Hall 2010) *intersect* to overlap the genomic coordinates of deleterious homozygous genotypes with ROH. To understand if genetic purging through inbreeding occurred at specific time frames (i.e., ancient past or more recently), we completed this analysis independently for ROH greater and < 2.5 Mb. We then calculated the proportion of deleterious homozygotes that were found within and outside of ROH for each individual. We preformed analyses separately for the autosomes and the X chromosome.

### 2.7 Mutation Load in the NARW

For interspecific analyses, we first generated a gVCF with only NARW using BCFtools filter ‐i and VCFtools filter ‐CHR, yielding 796,365 single nucleotide polymorphisms (SNPs) across the autosomes and 28,777 SNPs across the X chromosome. We then compared differences in mutation load between the female NARW with low and high estimates of RS to understand if genome wide variation could be linked to RS. Specifically, the low RS group was comprised of five adult females that have never been observed with calves and the high RS group was the five females with the highest number of observed calves to date (Table S1). To identify candidate variants linked to reduced RS in the NARW, we generated estimates of group‐level total mutation load for each site (the frequency of the weighted deleterious allele) and employed three outlier approaches. For the first of these we used the R package PCAdapt (Luu, Bazin, and Blum 2017), which utilizes dimension reduction and Malhalanobis distance to quantify the association of each variant with the overall pattern of genetic diversity (Luu, Bazin, and Blum 2017). PCAdapt then transforms the Malhalanobis distance for each variant to account for the inflation of genome‐wide statistics and to highlight outlier variants that do not align with stochastic processes (Luu, Bazin, and Blum 2017). For the second outlier approach, we estimated *F*_ST_ for all sites using VCFtools to quantify differences in genotype frequency between groups at each site. And for the third approach, we generated the per‐site difference in total mutation load (Δ total mutation load) between NARW females with high RS and those that are nulliparous. We opted to use total mutation load because deleterious alleles that are sheltered in heterozygote genotypes in mothers have some probability to become realized in calves, dependent on the genotype of the fathering male. Respectively, these approaches highlight alleles that (a) segregate deterministically, (b) are differentiated between groups, and (c) show a relatively high mutation load in nulliparous female NARW. We considered candidate variants to be those that were in the top 10 percentile in all three approaches. We visualized the number of variants for each approach and overlap between approaches using a web‐based Venn diagram generator available at https://bioinformatics.psb.ugent.be/webtools/Venn/.

We used two different gene enrichment databases implemented in the Webgestalt R package (Liao et al. 2019) to assess the functions across genes with candidate variants. First, we employed an over representation of gene ontology (GO) terms, selecting the top 10 results based on the highest enrichment ratios (the ratios of the observed counts to the expected counts given a particular gene set). Second, we used the Kyoto Encyclopedia of Genes and Genomes (KEGG) background, again selecting results based on the 10 highest enrichment ratios. To account for redundancy across terms, we used Webgestalt R to reduce GO Slim summary results to the minimum list of functional terms that could represent all genes with candidate loci. For instance, we generated histograms for categories of biological processes, cellular components, and molecular functions. For both enrichment approaches, we used the cow ( *Bos taurus* ) genome as a background gene set. We retained genes with multiple candidate variants because the accumulation of multiple mutations in a single gene increases the probability of important functional changes to that gene.

### 2.8 The Effects of Species, Genomic Region, and Predicted Mutation Impact

We employed Bayesian models to estimate the effects of species, genomic region, and predicted mutation impact (i.e., predictor variables) on patterns of genetic diversity, inbreeding, and mutation load (i.e., response variables). Specifically, we estimated the effect of each predictor variable level on the response variable, as well as the remaining variation not explained by predictor variables. Prior distributions were modeled from normal distributions with a mean of 0 and a standard deviation of 1, and response variables were *z*‐standardized. Prior distributions were checked to be informative by comparing prior distributions with the distributions of standardized response variables. Our models were written in Stan through the CmdstanR v2.32 interface (Stan Development Team 2017). Models were fit by running three chains in parallel, each with 10,000 MCMC iterations. For each model, we used a warmup of 1000 iterations. All models were checked for their performance and convergence by visualizing trace plots and assessing *rhat* values. The posterior distributions of all modeled parameters were then visualized for each model using ggplot2 in R (Wickham 2011).

## 3 Results and Discussion

Considering the history of commercial whaling (McLeod et al. 2008) and similar reproductive capabilities (Hamilton et al. 1998), it is intriguing that NARW do not reflect signs of population recovery similar to that of other *Balaenidae* whales. One hypothesis is that inbreeding depression is at least partially responsible for the lack of recovery in NARW. Here, our analyses of whole genome data reveal estimates of genetic diversity, inbreeding, and mutation load that align with this hypothesis (Table S2). However, we also show genomic evidence for the purging of deleterious alleles in NARW, which could increase the probability of future population recovery. Furthermore, we identified a suite of alleles putatively linked to embryonic development that segregate at low frequencies in NARW females with high RS and are carried at high frequencies in nulliparous adult females. These same alleles are nearly absent in SRW and BH (Table S3), suggesting that the accumulation of deleterious mutations linked to embryonic development in particular, might contribute to the absent or delayed population recovery of NARW.

### 3.1 Genetic Diversity and Inbreeding

**FIGURE 1:**
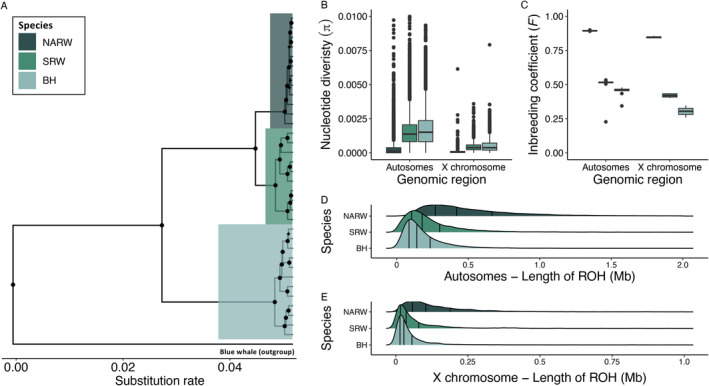
Estimates of genetic diversity and evolutionary relationships across *Balaenidae* whales. Species are colored as depicted in the legend. (A) Mitochondrial genome phylogeny estimated using BEAST. Posterior probabilities > 0.95 are denoted with a filled circle. (B) Estimates of nucleotide diversity within 100‐Kb windows across autosomes and the X chromosome. (C) Estimates of inbreeding given by F for autosomes and the X chromosome. Note that the order of species is consistent across (B) and (C). The distribution of the lengths of runs of homozygosity for each species for (D) the autosomes and (E) X chromosome. Error bars are (±) one standard error.

Our estimates of phylogenetic relationships across *Balaenidae* from whole mitochondrial genomes complemented previous topologies that used mitochondrial gene fragments, revealing three clades with right whales monophyletic to the BH whale (Malik et al. 2000; Rosenbaum et al. 2000) (Figure 1A). In addition, posterior distributions from Bayesian models estimating the impact of species on genetic variation (Figure S1) revealed interspecific differences in π and *F* that are compatible with previous estimates using mitochondrial gene fragments (Rosenbaum et al. 2000), microsatellite markers (Waldick et al. 2002), and autosomal variants (Crossman, Fontaine, and Frasier 2023) (Figure 1B,C). Notably, our estimates of π for NARW are much lower than that of either SRW or BH and place NARW within the lower tier of genetic diversity estimates across endangered mammals (as reviewed by Teixeira and Huber 2021).

Although low genetic diversity may not be casual of population decline, it can act as a meter for causal processes, such as inbreeding depression. It is thus central that our analyses also revealed values of *F* (Figure 1C) and ROH in NARW (Figure 1D,E) that are symptomatic of inbreeding (Pemberton et al. 2012; Ceballos et al. 2018). Our analyses of inbreeding parallel those of Crossman, Fontaine, and Frasier (2023) and include additional inbreeding statistics and metrics of ROH that we quantified across both autosomes and the X chromosome (Table 1). Because recombination disrupts long stretches of autozygosity, forming a greater number of shorter homozygous runs over an increasing number of generations, variation in the number and sum of ROH lengths can provide insight into historical demographic processes as well as contemporary patterns of inbreeding (Pemberton et al. 2012; Ceballos et al. 2018). We infer from the greater number of short ROH (i.e., < 100 Kb) and near absence of ROH of any length > 1 Mb in SRW and BH (Figures S2 and S3), that inbreeding in these populations has been uncommon throughout their histories. Within NARW, the high number of ROH between lengths of 1 Mb and 2.5 Mb suggests that inbreeding across more historical time frames was common, possibly due to a sustained small population size (Table 1). Meanwhile, the sharp decrease in the number of ROH > 2.5 Mb suggests that contemporary inbreeding is rare in NARW, considering that only ROH > 5 Mb reflect recent inbreeding (Ceballos et al. 2018). These results align with recent findings, that on average, the ROH observed within NARW formed approximately 500 generations ago and the most recent ROH formed between 12 and 25 generations ago (Crossman, Fontaine, and Frasier 2023).

**TABLE 1:** Estimates of runs of homozygosity (ROH) for *Balaenidae* species. Columns designate species, the number of ROH < 1 million base pairs, the number of ROH between 1 and 2.5 Mb, the number of ROH > 2.5 Mb, the number of ROH > 5 Mb, and the total number of ROH (NROH). Also shown are the mean length of ROH (Mean LROH), the total sum length of all ROH (SROH), the fraction of the genome captured in ROH (FROH), and the genomic region. Data are shown for the autosomes and for the X chromosome independently and values indicate the average value for individuals within each species.

Species	NROH < 1 Mb	NROH 2.5 Mb—1 Mb	NROH > 2.5 Mb	NROH > 5 Mb	NROH	Mean LROH	SROH	FROH	Genomic region
NARW	2396.2	279.4	17.8	3.8	2693.3	549,974.6	1,480,906,258	0.66	Autosomes
SRW	462.8	7.4	1.6	0.5	471.8	247,039.5	120,261,546	0.05	Autosomes
BH	2381.9	13.9	2.9	0.5	2398.7	191,787.9	459,921,240	0.21	Autosomes
NARW	509.6	2.5	0	0	512.10	150,645	76,878,340	0.60	X Chromosome
SRW	232.8	0	0	0	232.80	69,018	16,095,737	0.12	X Chromosome
BH	947.7	0.70	0	0	948.33	52,343	49,662,411	0.39	X Chromosome

### 3.2 Mutation Load

**FIGURE 2:**
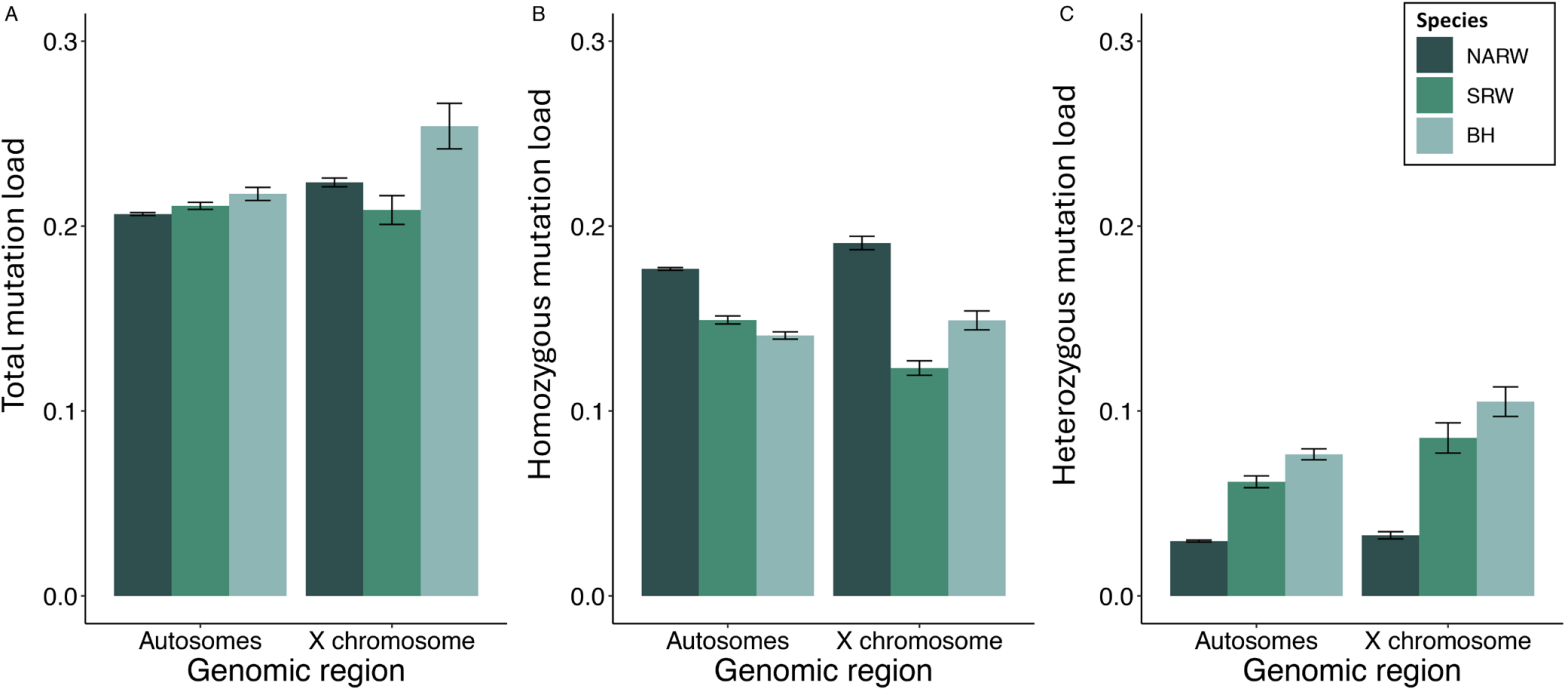
Estimates of mutation load across *Balaenidae* whales for autosomes and the X chromosome. Species are colored as depicted in the legend and genomic region is labeled along the X axis for (A) Total mutation load, (B) homozygous mutation load, and (C) heterozygous mutation load. Error bars are (±) one standard error.

We found similar estimates of total mutation load across NARW, SRW, and BH (Table S4). However, the homozygous mutation load, which represents the proportion of the realized mutation load caused by deleterious homozygotes, is higher in NARW, and the heterozygous mutation load, representing the masked mutation load, is higher in SRW and BH (Figure 2; Figure S4). The data used to generate estimates of mutation load can be visualized as derived allele and genotype frequencies per individual (Figures S5 and S6), as well as the unfolded SFS (Figures S7 and S8) (see also ‘Section 2’). The proportions of derived segregating and fixed alleles across each species also show a paucity of polymorphic sites in NARW and a higher number of sites segregating deleterious alleles at lower frequencies in SRW and BH (Figure S9). Reasonably, higher rates of inbreeding in NARW in the past because of small population size could have reduced the number of polymorphic sites over time, fixing most sites for either the ancestral or derived allele. An increase in the number of fixed sites would then most likely replace the heterozygous mutation load with a high homozygous mutation load (Kimura, Maruyama, and Crow 1963). Meanwhile, larger population sizes in SRW and BH could have led to increased heterozygous mutation loads relative to NARW. In these two species, larger population sizes would have allowed for greater number of meiotic events providing opportunity for germline mutations to enter the gene pool. The increased efficacy of selection associated with a larger population size would have then maintained deleterious variants at low frequencies. In tandem with estimates of demographic history (Crossman, Fontaine, and Frasier 2023), genetic diversity, and inbreeding, our estimates of mutation load are consistent with the hypothesis that inbreeding depression, albeit through more historical inbreeding, could partially account for the current recovery status of NARW.

### 3.3 Genetic Purging Through Inbreeding

In context of conservation, it is important to highlight that a number of populations have survived severe declines despite the associations between small population size, inbreeding depression, and extinction (Frankham 1998). Potentially, survival in such cases has been underpinned by selection against recessive deleterious alleles that were unmasked in homozygous genotypes because of inbreeding. The loss (i.e., purging) of deleterious alleles from such declining populations could have slowed or stopped population decline (as in Robinson et al. 2018).

**FIGURE 3:**
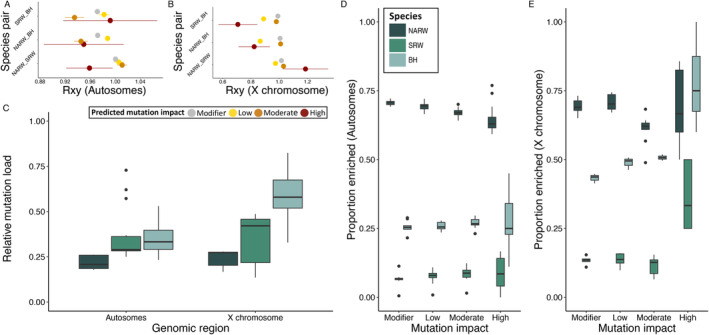
Genetic purging across *Balaenidae* whales. (A) Mean values of *Rxy* for the autosomes and (B) for the X chromosome. Note that posterior probability estimates are shown in Figure S10 in Supporting Information. The predicted mutation impact is colored as depicted in the legend. (C) The relative mutation load (homozygous mutation load/*F*) for both autosomes and the X chromosome for each population. The proportion of deleterious homozygotes found within runs of homozygosity for the autosomes and (D) for the X chromosome (E) for each predicted mutation impact. Species are colored as depicted in the legend. Error bars are (±) one standard error.

A growing number of genomic studies are inferring genetic purging according to observed deficits of high impact alleles that are accompanied by surpluses of mildly deleterious alleles in bottlenecked populations relative to larger populations (e.g., Xue et al. 2015; Grossen et al. 2020; Kleinman‐Ruiz et al. 2022). Our analyses of deleterious allele frequencies uncover patterns in *Rxy* of varying predicted mutation impacts (Figure 3A,B; Figure S10) that are similar to those of other mutation load studies, including a recent assessment of inbreeding depression in killer whales ( *Orcinus orca* ) (Kardos et al. 2023). Specifically, the frequency of high impact alleles in our autosomal dataset is reduced in NARW relative to SRW despite the increased frequencies of low and moderate impact deleterious alleles in NARW (Figure S11). This pattern is consistent with theoretical expectations of genetic purging, where the efficacy of selection is proportional to population size, allowing lower impact mutations to accumulate to higher frequencies in smaller populations (Ohta 1973). At the same time, highly deleterious recessive mutations (i.e., “recessive lethals”) are simultaneously exposed to selection in homozygous genotypes in bottlenecked or inbred populations (Kimura, Maruyama, and Crow 1963), leading to the purging of high impact deleterious alleles despite the accumulation of lower impact deleterious alleles (Dussex et al. 2023). Notably, although the frequency of autosomal modifier alleles (i.e., alleles inferred to incur in noncoding regions) is similar between NARW and SRW, supporting annotations through SnpEff, there appears to be a deficit of all impact categories, including modifier alleles for both NARW and SRW compared to BH. Because modifier impact mutations are anticipated to segregate under neutrality, this pattern feasibly stems from the increased divergence time between the *Eubalaena* and *Balaena* genera, allowing for greater gene turnover and back mutation (Arbogast et al. 2002).

To interrogate genomes for signs of genetic purging *through inbreeding*, we tested relationships between the frequency of deleterious homozygous genotypes and inbreeding statistics. Our approaches hinge on expectations that the rate at which the homozygous mutation load replaces the heterozygous mutation load corresponds with levels of inbreeding (Barrett and Charlesworth 1991). Two recent studies concerned with declining populations of Bengal tigers ( *Panthera tigris tigris* ) (Khan et al. 2021) and killer whales (Kardos et al. 2023) empirically validated this expectation with positive correlations between FROH and the homozygous mutation load. The enrichment of deleterious mutations in ROH has also been shown in humans ( *Homo sapiens* ) (Szpiech et al. 2013).

When accounting for estimates of *F*, we found an unexpectedly low homozygous mutation load (relative mutation load) in NARW (Figure 3C; Figure S12). This suggests that inbreeding has had an attenuated effect on replacing the heterozygous mutation load with the homozygous mutation load in NARW relative to SRW and BH, plausibly resulting from the purging of homozygous genotypes from within regions of the genome linked to inbreeding. We explored this idea further by comparing the proportion of homozygous deleterious genotypes inside and outside of ROH. According to theory, homozygous genotypes should occur at a frequency of *p* ^2^ outside of ROH and a frequency of *p* inside ROH (Szpiech et al. 2013). However, if genetic purging occurs through inbreeding, then the frequency of deleterious alleles should be reduced within ROH. A clear pattern of this expected reduction was observed within NARW, but not for SRW or BH (Figure 3D,E). We found a decreasing enrichment of deleterious homozygous genotypes in ROH across mutation impact categories that scaled according to expected selection coefficients. Specifically, the enrichment of high impact mutations is reduced compared to modifier alleles in NARW across autosomes (Figure S13). In context of *Rxy* and the relative mutation load, this pattern indicates that genetic purging *through inbreeding* has contributed to shaping deleterious allele frequencies in NARW, at least across autosomes. Still, it is important to restate that the loss of deleterious alleles in NARW would have occurred in the past through more ancient inbreeding events likely due to sustained small population size. Indeed, the increased loss of high impact deleterious alleles compared to modifier alleles in NARW ROH does not hold for ROH > 2.5 Mb across the autosomes (Figure S14).

### 3.4 Variation Between Genomic Regions

Although the overall similarity of mutation load and derived allele frequencies across genomic regions could imply that the efficacy of genetic purging through inbreeding is similar between the autosomes and the X chromosome, we did find important variation within estimates of genetic diversity, inbreeding, and mutation load that is feasibly linked to hemizygosity. Beyond expected reductions in π (Figure 1B; Figure S15) and differences in ROH statistics (Table 1; Figure S16), which have been linked to the absence of homologous recombination (Ellegren 2009), we also found that the relative mutation load was greater for the X chromosome compared to the autosomes (Figure 3B; Figure S17). Counter to the conclusions of Kleinman‐Ruiz et al. (2022), this suggests that inbreeding can have a marked impact on the homozygous mutation load of hemizygous sex chromosomes. Moreover, estimates of *Rxy* (for high impact derived alleles) for both right whales relative to BH are lower for the X chromosome than for the autosomes (Figure 3B), which could be due to the increased efficacy of genetic purging (Smeds and Ellegren 2023). Nevertheless, a greater efficacy of genetic purging through inbreeding on the X chromosome would be expected to reduce (not increase) the relative mutation load compared to the autosomes. In addition to mutation load, we also found that genomic region had little effect on estimates of *Rxy* or and the proportion of deleterious alleles enriched in ROH (Figure S18). These results might suggest that the fate of deleterious variation on the X chromosome is influenced by accelerated drift (Chen et al. 2018); yet still, other features of hemizygosity should be considered.

In addition to the increased exposure of recessive variation to selection and a lack of homologous recombination in the heterogametic sex (Morgan 1912), hemizygosity also results in a smaller effective population size (*N*_*e*_) for sex chromosomes (Mank et al. 2010), reduced mutation rates (Haldane 1946; Charlesworth 1978), intralocus sexual conflict (Manat et al. 2021), and dosage compensation (Charlesworth 1978). Complex relationships among these mechanisms of molecular evolution are more than likely to have nuanced impacts on the frequency of deleterious variation across the X chromosome that may be spatially and temporally dependent (Mank et al. 2010; Wilson Sayres 2018). For example, the homozygous mutation load is reduced on the X chromosome relative to the autosomes for SRW, but not for NARW or BH (Figure 2B). We also note that moderate impact (but not high impact) deleterious alleles are less enriched in ROH compared with modifier and low impact mutations across the X chromosome for NARW and SRW (Figure 3D,E). Meanwhile, the enrichment of high impact deleterious alleles is described by a large degree of variation for all three species. There are also deficits in the number of low impact alleles in both right whales compared to BH for the X chromosome (Figure 3B). Patterns such as these could result from the faster adaptive evolution of the X chromosome (Charlesworth, Campos, and Jackson 2018), where the selective impact of alleles vary across species. Structural rearrangements of the X chromosome have occurred at least five times during the evolution of Artiodacytla (Proskuryakova et al. 2017), signifying the potential for rapid evolution of the X chromosome within whales.

Our independent analyses of different genomic regions support previous conclusions that hemizygosity leads to unusual patterns of evolution on the X chromosome (as reviewed by Vicoso and Charlesworth 2006). A detailed experiment with large sample sizes of both males and females accounting for the Pseudo Autosomal Region is needed to disentangle the relationships between hemizygosity and mutation load. Here, we were most concerned with understanding the potential impact of inbreeding and mutation load on recovery potential of NARW. As such, genomic resources were focused on additional females because the effects of a deleterious allele on the mean fitness of a population largely depends on its effect on female RS (Whitlock and Agrawal 2009), and female RS disproportionately impacts *N*_*e*_ (Nunney 1996). Decades of life history data collected through the ability to photographically identify individual NARW (Hamilton, Knowlton, and Marx 2007) show that females exhibit substantial variation in estimated RS despite shared habitat (Hamilton, Knowlton, and Marx 2007), suggesting the impact of intrinsic factors. We therefore sequenced the genomes of females with the highest and lowest estimates of observed calving events since the early 1980s to more thoroughly assess the impact of deleterious genetic variation on NARW population recovery.

### 3.5 Mutation Load and Reproductive Success in NARW

**FIGURE 4:**
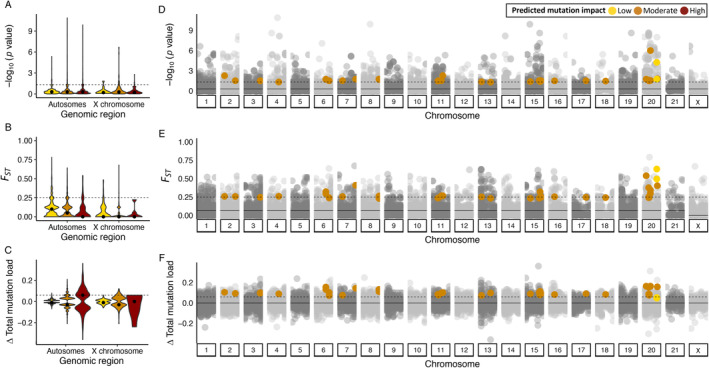
Deleterious mutations across the genomes of adult female NARW for the autosomes and the X chromosome. Violin plots summarizing variation are shown to the left and associated Manhattan plots for each outlier approach are shown to the right. Genomic region and position are labeled accordingly along the X axes. The predicted impact of nonsynonymous mutations is colored as depicted in the legend. Note that only the 41 variants found to be outliers across all three approaches are highlighted in Manhattan plots. Gray points in Manhattan plots reflect nonsynonymous mutations that were not classified as candidate variants. Median values are indicated with solid circles or lines and the assigned outlier thresholds are shown as dotted lines. (A) The distribution of −log_10_ *P*‐values for variant alignment with Principal Components and (B) Manhattan plot of the −log_10_ *P*‐values for variant alignment with Principal Components. (C) The distribution of *F*_ST_ values per site between the genomes of high fecund and nulliparous NARW. (D) Manhattan plot of *F*_*ST*_ values per site between the genomes of high fecund and nulliparous NARW. (E) The distribution of differences in total mutation load per site between the genomes of high fecund and nulliparous NARW females and (F) Manhattan plot of differences in total mutation load per site between the genomes of high fecund and nulliparous NARW females. Positive values indicate a greater mutation load in nulliparous NARW.

We did not find differences in genome‐wide estimates of mutation load or inbreeding between NARW with high and low estimates of RS (Figure S19). However, we did identify deleterious variants putatively associated with nulliparity in NARW females using approaches similar to those used to assess the genetic effects of inbreeding in the Isle Royale wolf ( *Canis lupus* ) (Robinson et al. 2019) and to explore positive selection in the Ethiopian wolf (*Canis simenis*) (Mooney et al. 2023). In our dataset, 41 variants were distributed across the top 10 percentiles of each of three candidate approaches based on associations with principal components, genotypic differentiation (*F*_*ST*_), and differences in total mutation load (Figure 4A–F; Figure S20). Respectively, these approaches highlight variants that are outlier to the genomic background, segregate at different frequencies in females with high and low RS, and are putatively deleterious. The 41 candidate variants are therefore expected to represent a suite of mutations that collectively impart a high mutation load in nulliparous females but segregate at low frequencies in females with high RS due to purifying selection that has yet fully purged deleterious variation from NARW.

**FIGURE 5:**
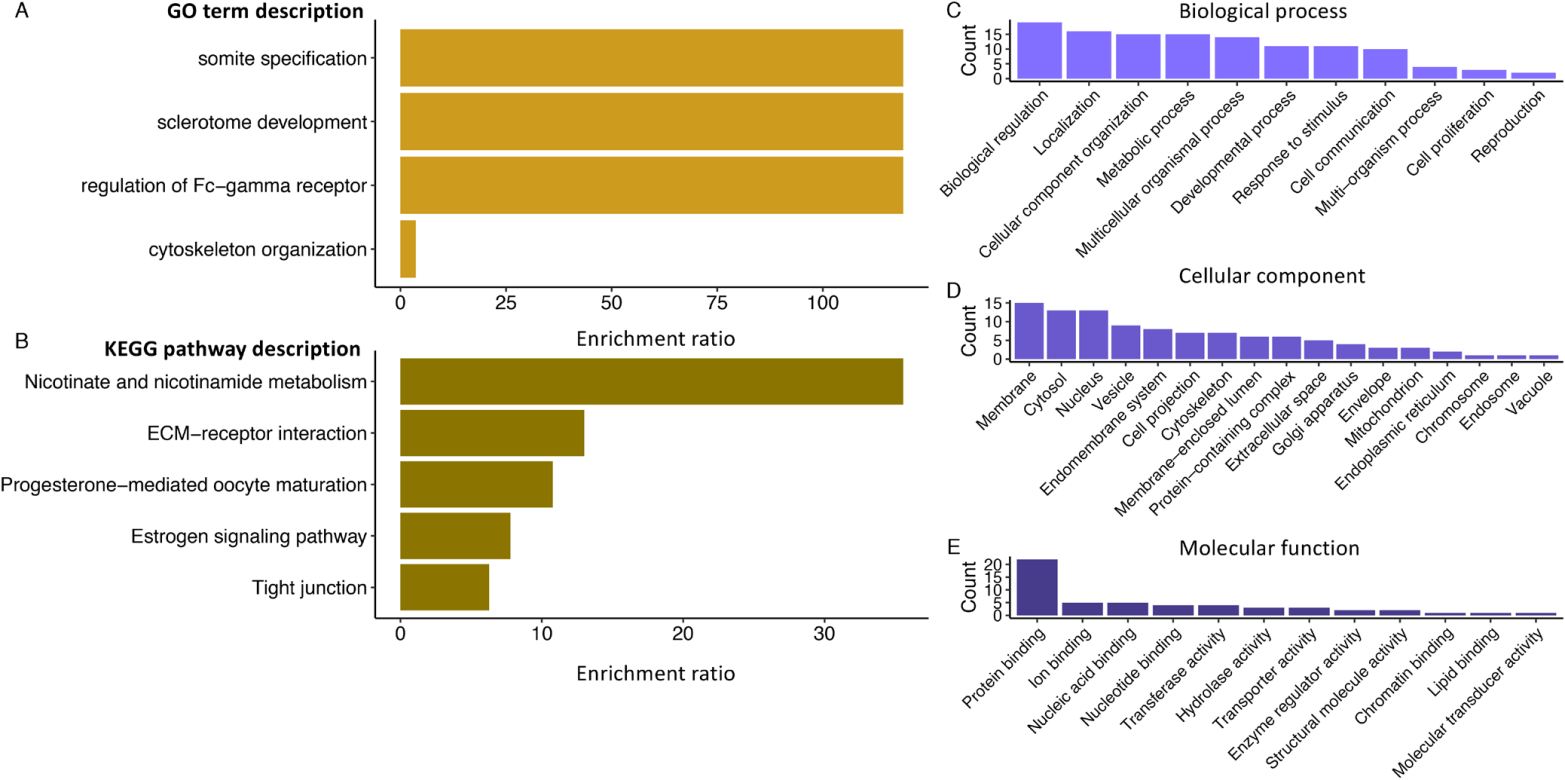
Results of gene enrichment analyses showing only weighted terms to account for redundancy. (A) Nonredundant terms with the highest gene enrichment values from over‐representation analysis using a Gene Ontology background. (B) Nonredundant terms with the highest gene enrichment values from over‐representation analysis using the KEGG background. GO‐Slim summary terms for biological process (C), cellular component (D), and molecular function (E).

Using two gene enrichment analyses, we found that the most over‐represented functional terms among candidate loci were primarily associated with embryonic development and protein binding across cellular membranes (Figure 5A–E). For instance, two of the top three over‐represented GO terms were “somite specification” and “sclerotome development,” critical facets of vertebral column development during embryogenesis (Tani et al. 2020). This is interesting with respect to the highly inbred Isle Royale wolf, where 58% of the population was documented with congenital defects of the lumbosacral vertebrae (Räikkönen et al. 2009). Over‐represented KEGG pathway terms complemented GO enrichment terms, where “Nicotine and Nicotinamide metabolism” (nicotinamide adenine dinucleotide [NAD+]) had the highest enrichment ratio. Notably, NAD+ has been associated with congenital defects and miscarriages in both human and mouse ( *Mus musculus* ) (Shi et al. 2017), and it plays a crucial role in oocyte development in a variety of livestock, including cattle (Pollard et al. 2022). The other over‐represented terms were also linked to development (e.g., “progesterone‐mediated oocyte maturation”) or Fc Gamma receptors, which are involved in pathogen recognition (Junker, Gordon, and Qureshi 2020). Note that all redundant terms are shown in Figure S21.

The effects of inbreeding depression have been documented to manifest early in life, often as developmental defects or juvenile mortality (Charlesworth and Willis 2009). In one stock of cattle, for example, inbred calves were born underdeveloped with descriptions akin to Fanconi–Bickel syndrome, an autosomal recessive disorder in humans (Pausch et al. 2015). More importantly, these inbred calves were homozygous for mutations to SLCA2A that cause Fanconi–Bickel syndrome in humans (Santer et al. 2002; Pausch et al. 2015). This is analogous to Isle Royale wolves, where individuals with vertebral abnormalities were found homozygous for mutations to an ortholog of SLC52A2, a gene associated with “abnormality of the vertebral column” in humans (Robinson et al. 2019). A recent study of NARW also found that perinatal mortality was likely linked to developmental abnormalities (Sharp et al. 2019). In our study, three of the 41 candidate variants mapped to two SLC (solute carrier—membrane transport) genes, while others mapped to genes such as NSUN5 and MEOX1, which are respectively linked to human developmental disorders (Heissenberger et al. 2019) and impact somite formation in chicken (*Gallus domesticus*) (Reijntjes, Stricker, and Mankoo 2007), zebra fish ( *Danio rerio* ) (Nguyen et al. 2014), and mouse (Mankoo et al. 2003). Similar to Isle Royale wolves (Robinson et al. 2019), only three of the 41 candidate variants were present in SRW and all were absent in BH (i.e., healthy individuals) (Table S3).

Although we present our results with caution due to limited sample sizes and the assumptions inherent of mutation load and gene enrichment analyses, we also emphasize that our results reinforce findings from two previous genetic studies suggesting that perinatal mortality in NARW is biased towards homozygous genotypes (Frasier et al. 2013; Crossman, Fontaine, and Frasier 2023). First, a study based on 28 microsatellite loci across 105 NARW mother–father–calf triads found that observed calves where more heterozygous than expected given the parental genotypes (Frasier et al. 2013). One explanation for these “missing homozygotes” was that inbred calves did not survive to term. More recently, genomic data (ddRADSeq analyses of ~100 females) showed that for all individuals, observed heterozygosity was higher than expected, again suggesting the biased loss of homozygous calves (Crossman et al. 2024). Collectively, our results indicate the presence of genetic purging due to a sustained small population size that may occur through selection against congenital defects. Undetected pregnancies and calving events could then account for the increased inter‐calf interval and high frequency of nulliparous NARW females (Pettis, Pace III, and Hamilton 2021) if such events conclude in perinatal mortality as previously hypothesized from life history data in NARW (Browning, Rolland, and Kraus 2009).

## 4 Conclusion

Since divergence within *Balaenidae*, both SRW and BH have persisted with larger *N*_*e*_ than NARW (Cerca et al. 2022; Crossman, Fontaine, and Frasier 2023). Note that both Cerca et al. (2022) and Crossman, Fontaine, and Frasier (2023) used Stairwayplot (Liu and Fu 2020) to estimate historical changes in *N*_*e*_ and thus, estimates from these studies should be generally comparable. With respect to mutation load, the stronger selection (and decreased genetic drift) associated with larger *N*_*e*_ (Kimura, Maruyama, and Crow 1963; Ohta 1973) could have maintained deleterious alleles with non‐lethal effects at lower frequencies in SRW and BH, while the same mutations appreciably accumulated in NARW under relaxed selection (Henn et al. 2016; Cui et al. 2019). Because the dominance of mutations is expected to scale inversely with the level of “deleteriousness” (Wright 1929; Gillespie 1977), there may be increased opportunity for non‐lethal mutations to impart gradual and synergistic fitness losses while accumulating in declining populations (Agrawal and Whitlock 2012). The importance of low impact deleterious genetic variation has been previously described (Kimura, Maruyama, and Crow 1963; Ohta 1992), and each of the 41 candidate variants found in this study was annotated as either low or moderate impact. We reiterate that only three of these candidate variants were present across the two *Balaenidae* species with sustained large historical *N*_*e*_. Similar to theoretical simulations and empirical data in Chinese crocodile lizards ( *Shinisaurus crocodilurus* ) (Xie et al. 2022) and fin whales (*Balaenotera physalus*) (Nigenda‐Morales et al. 2023), differences in historical population sizes could have shaped the observed variation in mutation load and population recovery across *Balaenidae* whales.

## 5 Implications for Conservation

The extent to which genetic diversity affects extinction and population recovery has been a source of contention for decades (Frankel and Soulé 1981). Genetic factors, for example, do not impact habitat fragmentation, climate change, or the pollution of natural environments. The genetic parameters of populations, such as mutation load, can however, unequivocally contribute to population status (Hohenlohe, Funk, and Rajora 2021). In NARW, the reduced population recovery is attributed to mortality from vessel strike and non‐lethal entanglement in fishing gear, as well as to a reproductive rate three times lower than the estimated potential (Moore et al. 2021). Although deleterious genetic variation may not impact the chances of anthropogenic injury, reduced reproductive rates are a hallmark of high mutation load (as reviewed by Agrawal and Whitlock 2012). Potentially, the estimates of mutation load quantified here can be used in tandem with estimates of RS to refine models of expected NARW population recovery. For example, recent models have utilized mutation load estimates to more accurately project recoveries in the critically endangered Vaquita ( *Phocoena sinus* ) (Robinson et al. 2022) and the threatened southern resident killer whale (Kardos et al. 2023). Currently, we are working to incorporate these genetic data into population viability models that will then be used to aid population management. These data are anticipated to have implications for setting appropriate goals and expectations, including the number of allowable “takes.”

## Conflicts of Interest

The authors declare no conflicts of interest.

## Supporting information


Figure S1.

Table S1.

## Data Availability

Right whale genomic data are available through NCBI's Sequence Read Archive under accession numbers SRR22863734—SRR22863755 (BioProject: PRJNA914998). Genomic data for bowhead whale are available through NCBI's Sequence Read Archive under Bioproject: PRJNA643010 and Bioproject: PRJNA798027. The code used in this manuscript, as well as example data and Bayesian models are available at https://github.com/richardorton/NARW_MutationLoad.
