# Supplementary material for: Genomic Evidence for the Purging of Deleterious Genetic Variation in the Endangered North Atlantic Right Whale
Source: Evol Appl. 2024 Dec 23;17(12):e70055. doi: 10.1111/eva.70055 (PMC11665784; doi:10.1111/eva.70055)
Supplement: Supplementary file 1 — Figure S1. [file EVA-17-e70055-s002.pdf]

Supporting Information for:

Genomic Evidence for the Purging of Deleterious Genetic Variation \in the Critically Endangered North Atlantic Right Whale

Richard W. Orton[1,*]
https://orcid.org/0000-0003-4028-248X

Philip K. Hamilton[2]

Timothy R. Frasier[1]

Corresponding author email: Richard.orton@smu.ca

This PDF file includes:
Figures S1 to S21

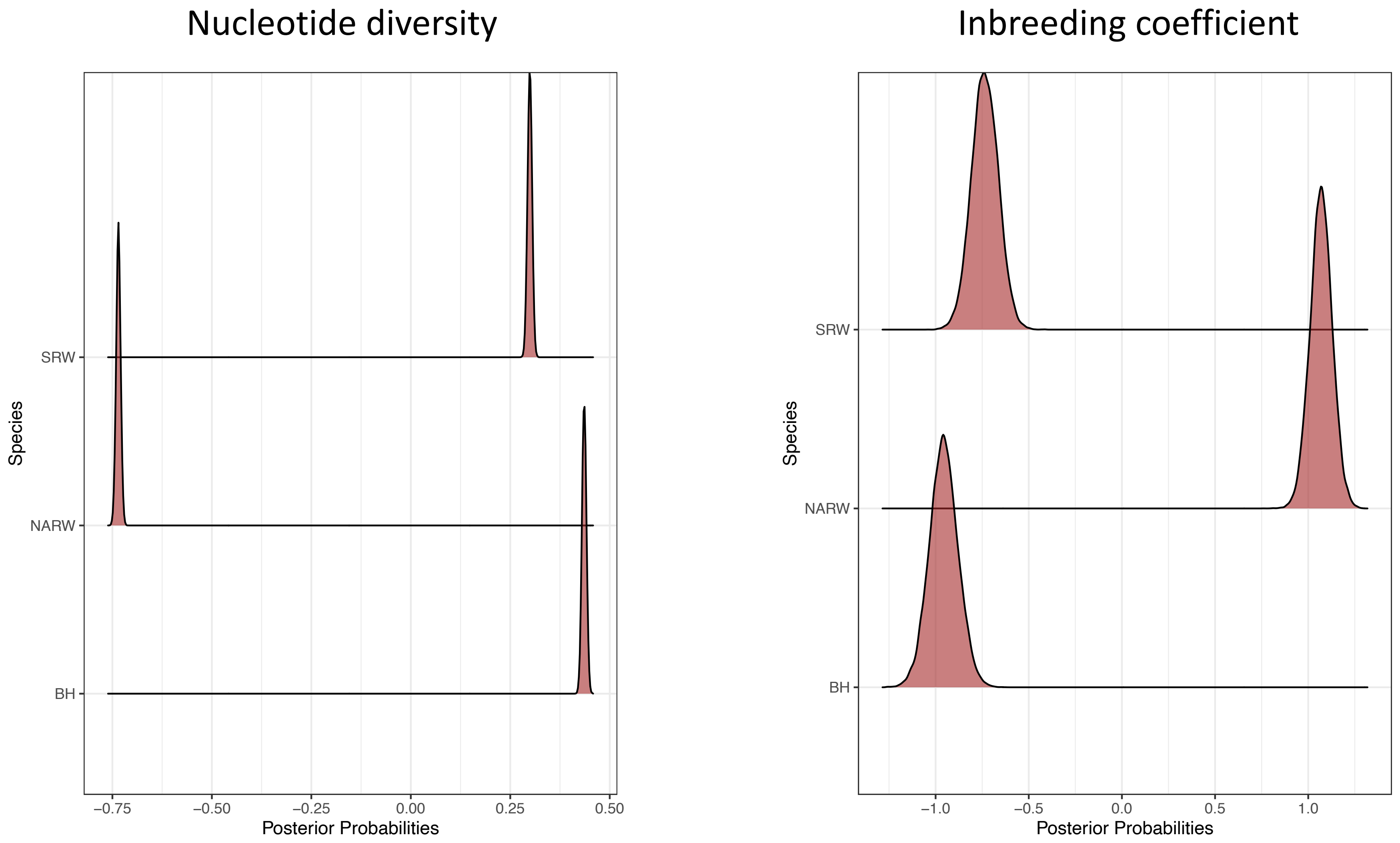

Fig. S1. Posterior probability distributions of the effect of species on standardized nucleotide diversity and the inbreeding coefficient.

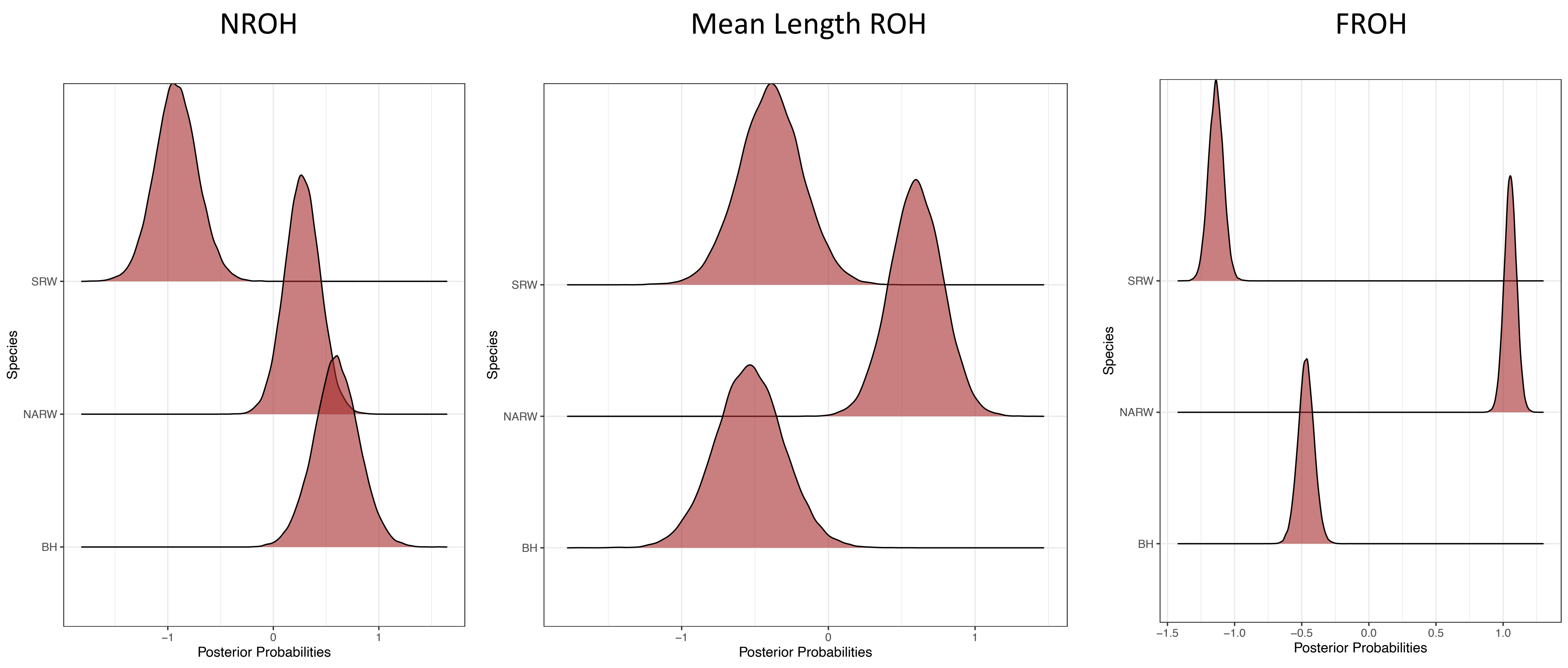

Fig. S2. Posterior probability distributions for the the effect of species on the standardized number and length of runs of homozygosity, as well as the fraction of the genome captured within runs of homozygosity.

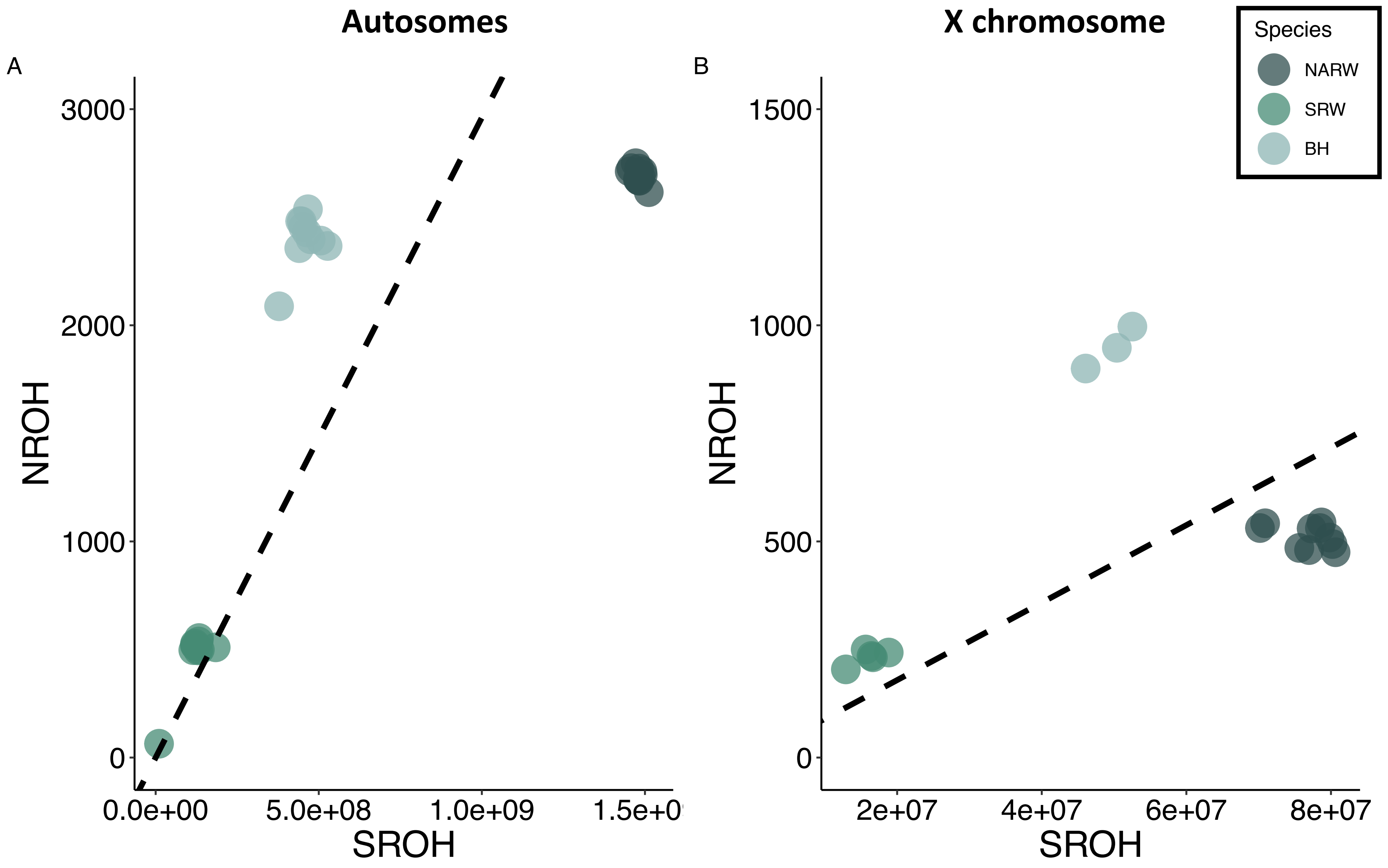

Fig. S3. The relationships between the number and total sum length of runs of homozygosity for the autosomes and the X chromosome. The dashed line indicates the hypothetical relationship between NROH and SROH given a constant length of ROH designated as the mean length of ROH across *Balaenidae* for the autosomes and the X chromosome independently.

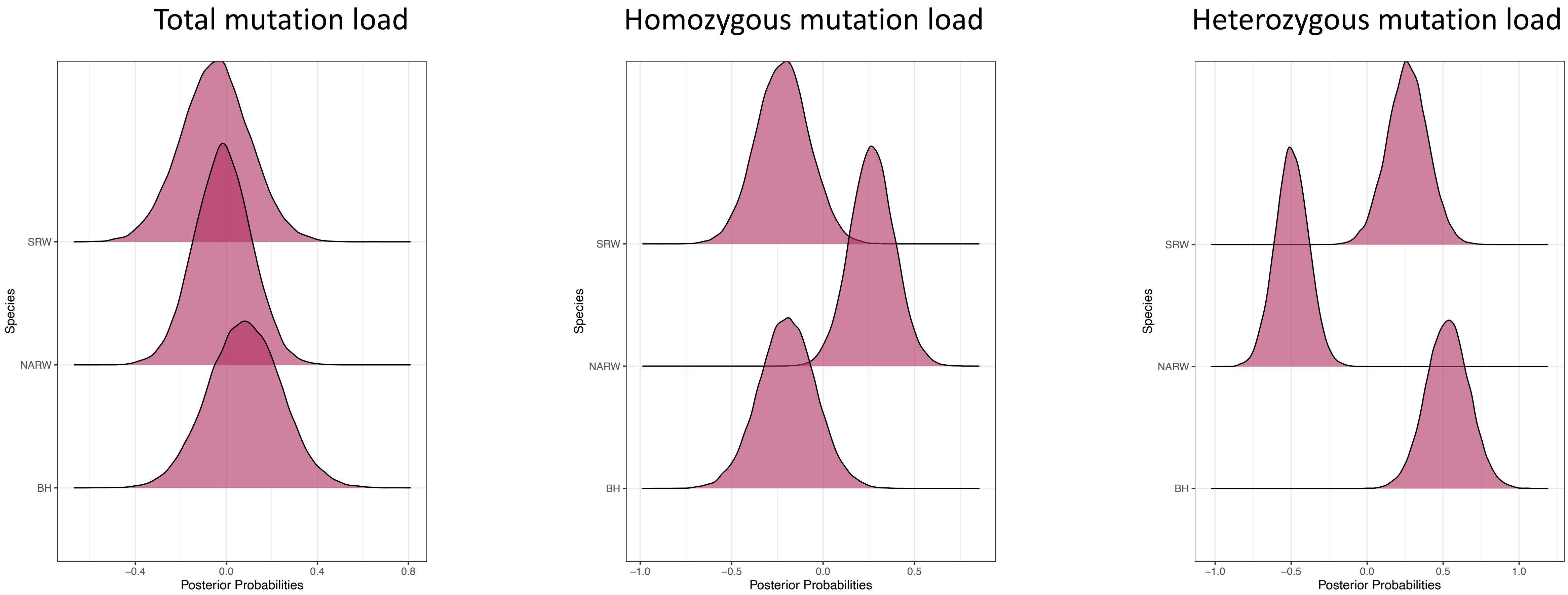

Fig. S4. Posterior probability distributions for the relationship between species and standardized mutation load.

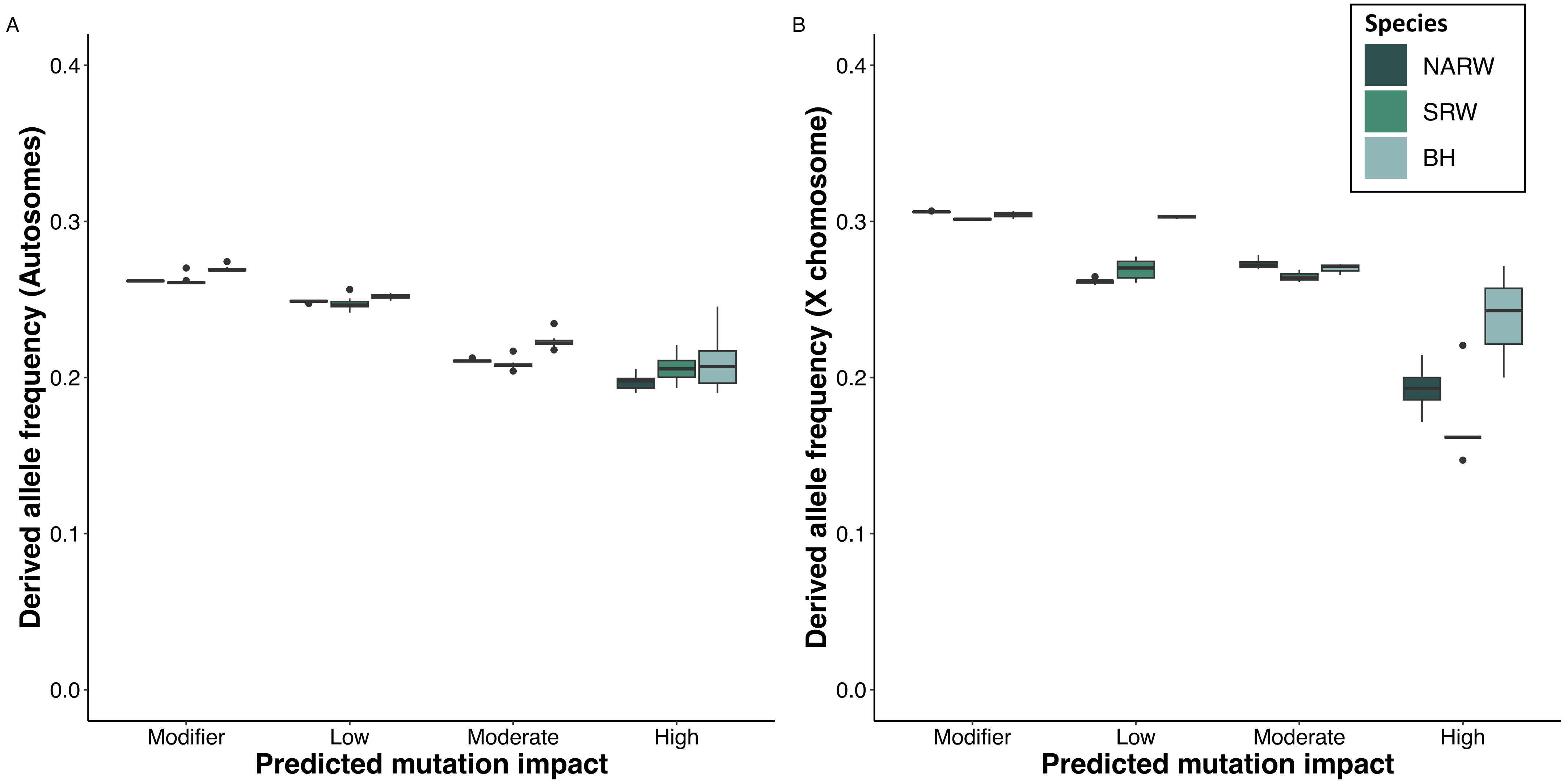

Fig. S5. Derived allele frequencies for NARW, SRW, and BH for the autosomes and the X chromosome. Note that these are the allele frequencies used in our analyses of mutation load (Figure 2) and Rxy (Figure 3). Allele frequencies have been generated for each predicted mutation impact category independently. Error bars are (+/-) one standard error.

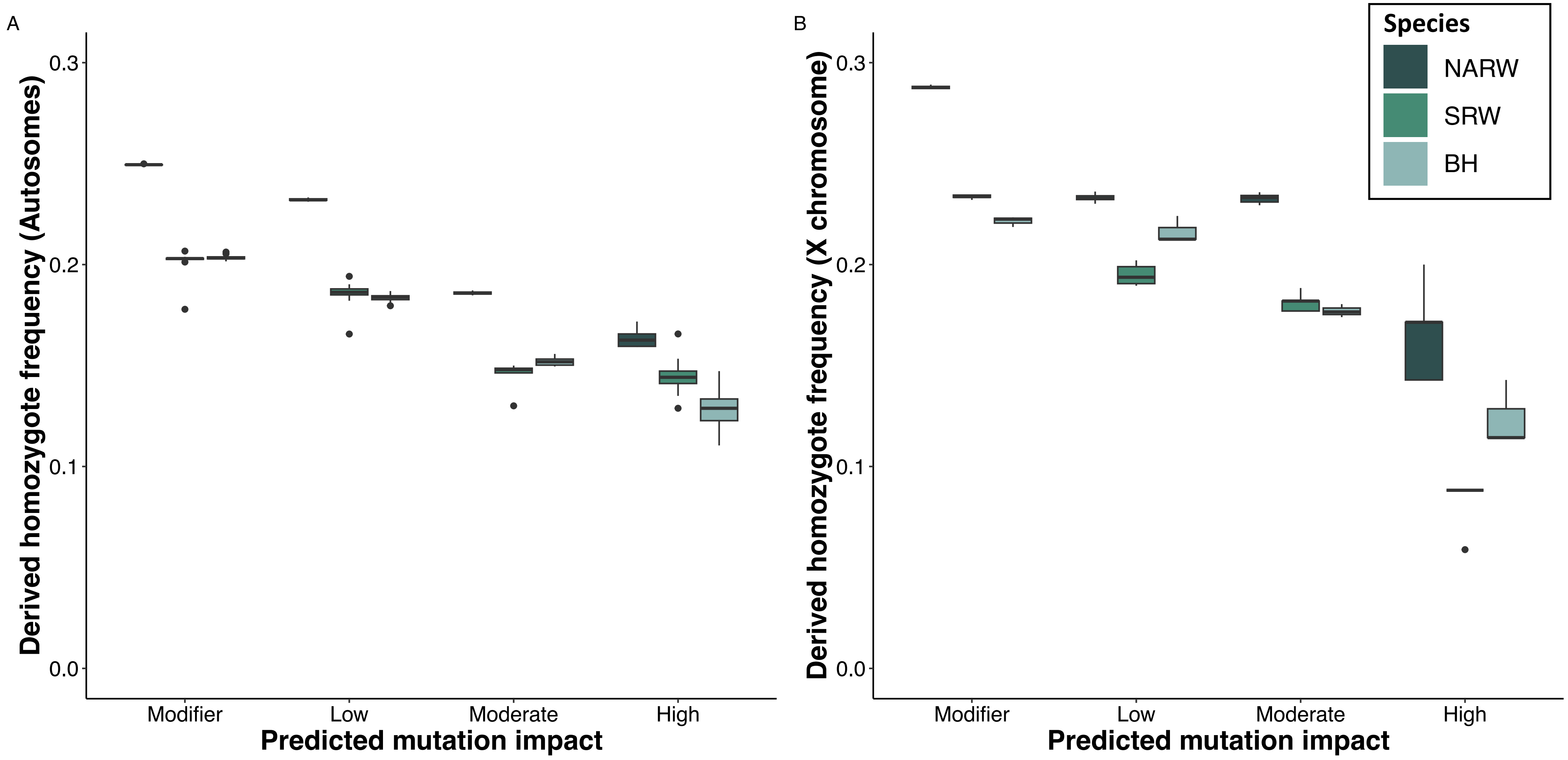

Fig. S6. Derived homozygous genotype frequencies for NARW, SRW, and BH for the autosomes and the X chromosome. Homozygous genotype frequencies have been generated for each predicted mutation impact category independently. Note that these are the genotype frequencies used in our analyses of mutation load and Rxy (Figure 2). Error bars are (+/-) one standard error.

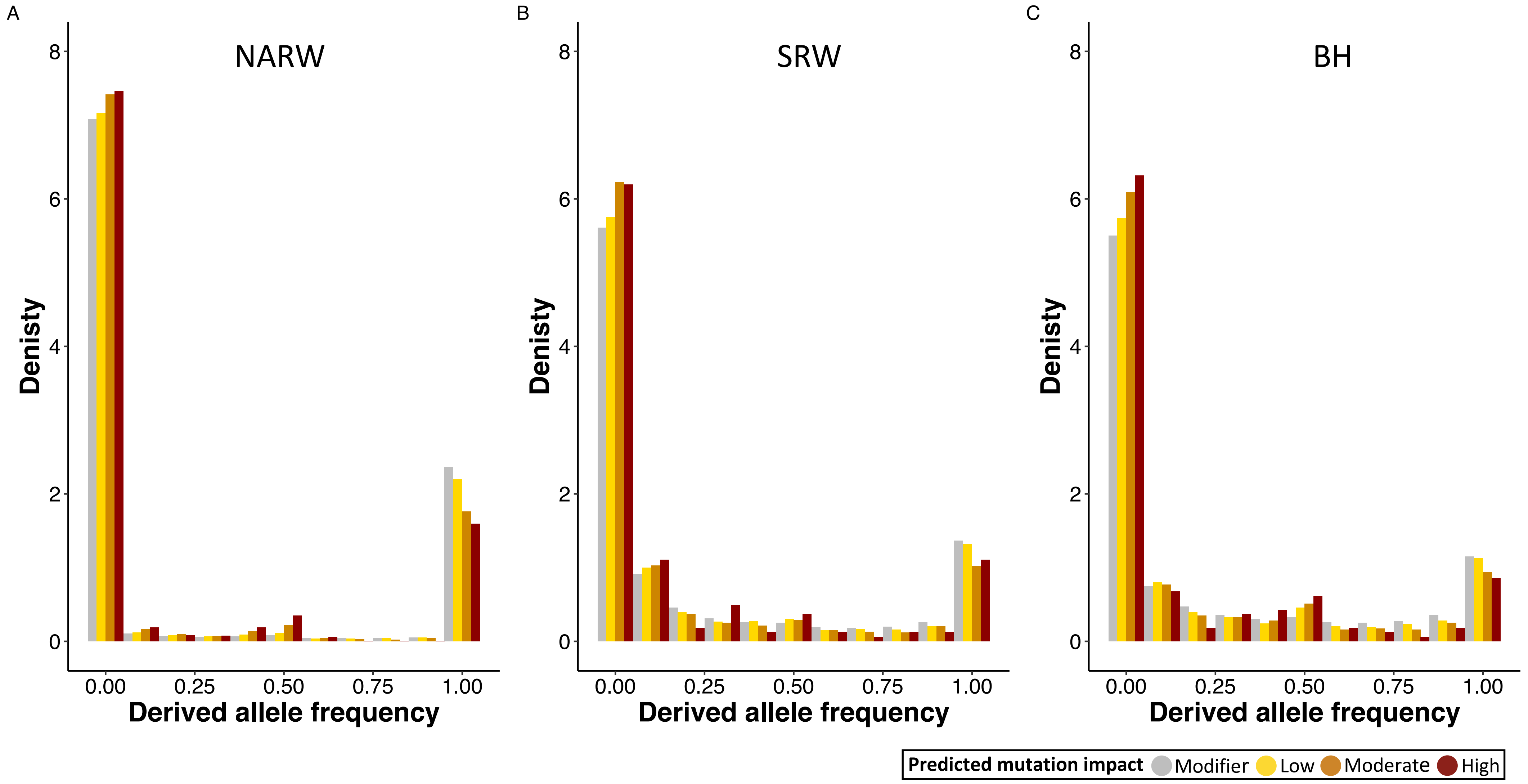

Fig. S7. The unfolded site frequency spectra for NARW, SRW, and BH for the autosomes. Spectra have been generated for each predicted mutation impact category independently.

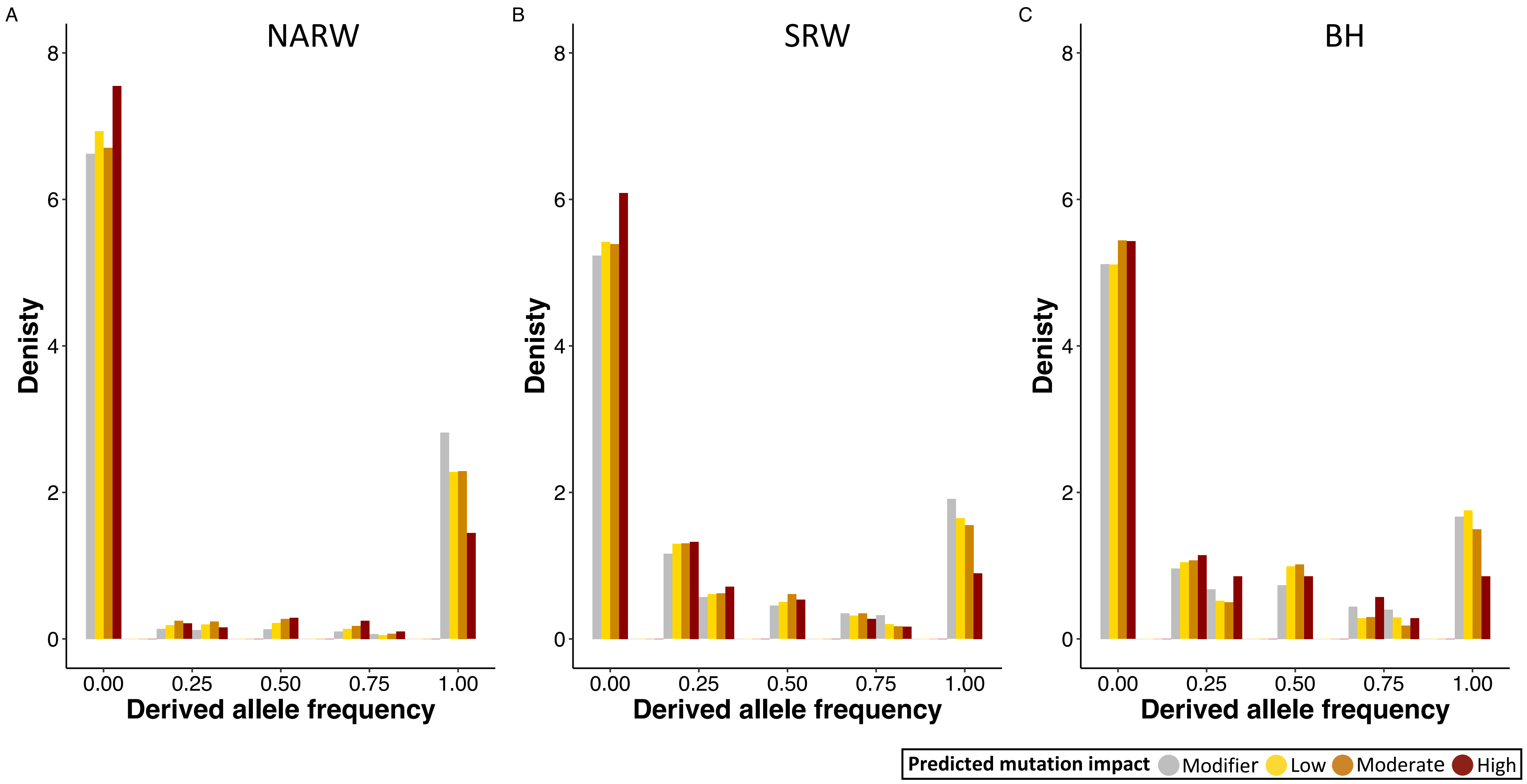

Fig. S8. The unfolded site frequency spectra for NARW, SRW, and BH for the X chromosome. Spectra have been generated for each predicted mutation impact category independently.

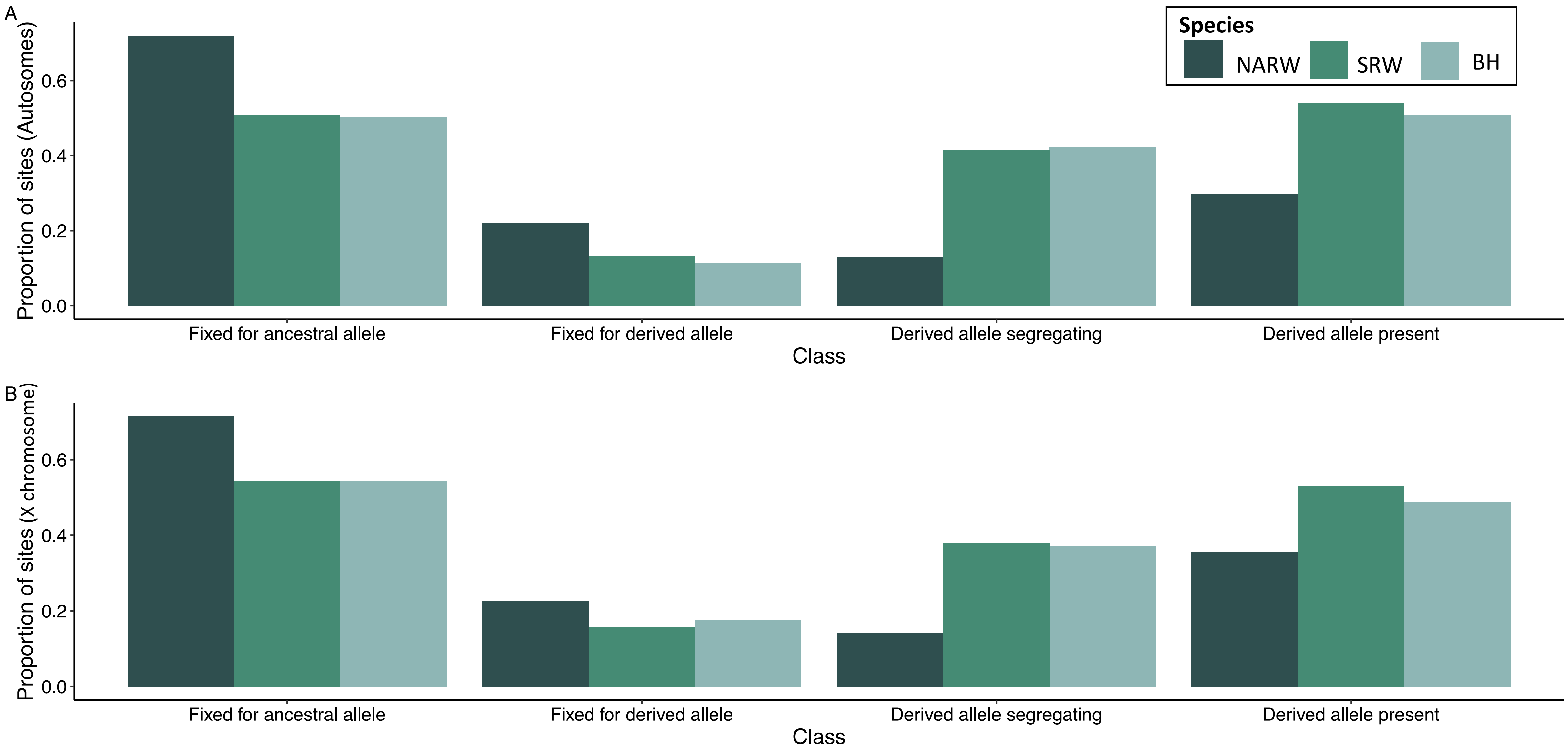

Fig. S9. The proportion of sites either fixed or segregating ancestral and derived alleles for NARW, SRW, and BH for the autosomes and the X chromosome. Also shown are the proportion of sites either fixed or segregating the derived allele. Note that the data shown here are derived from the total counts for each species.

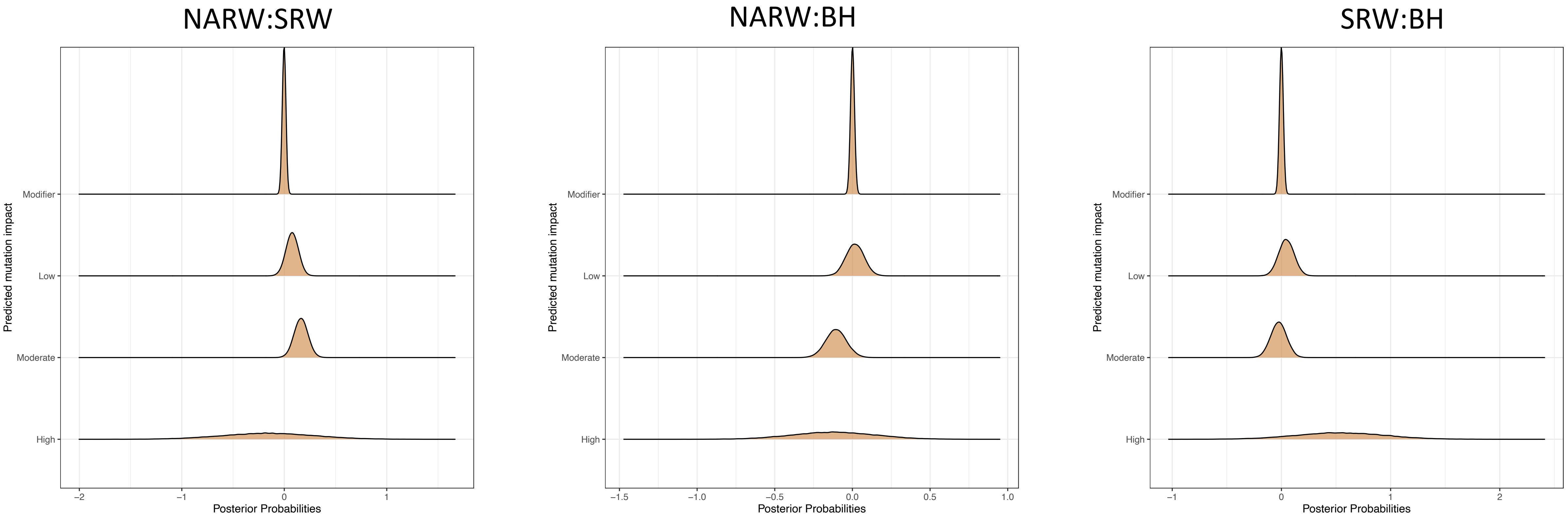

Fig. S10. Posterior probability distributions for the effect of predicted mutation impact category on standardized *Rxy* for each species pair.

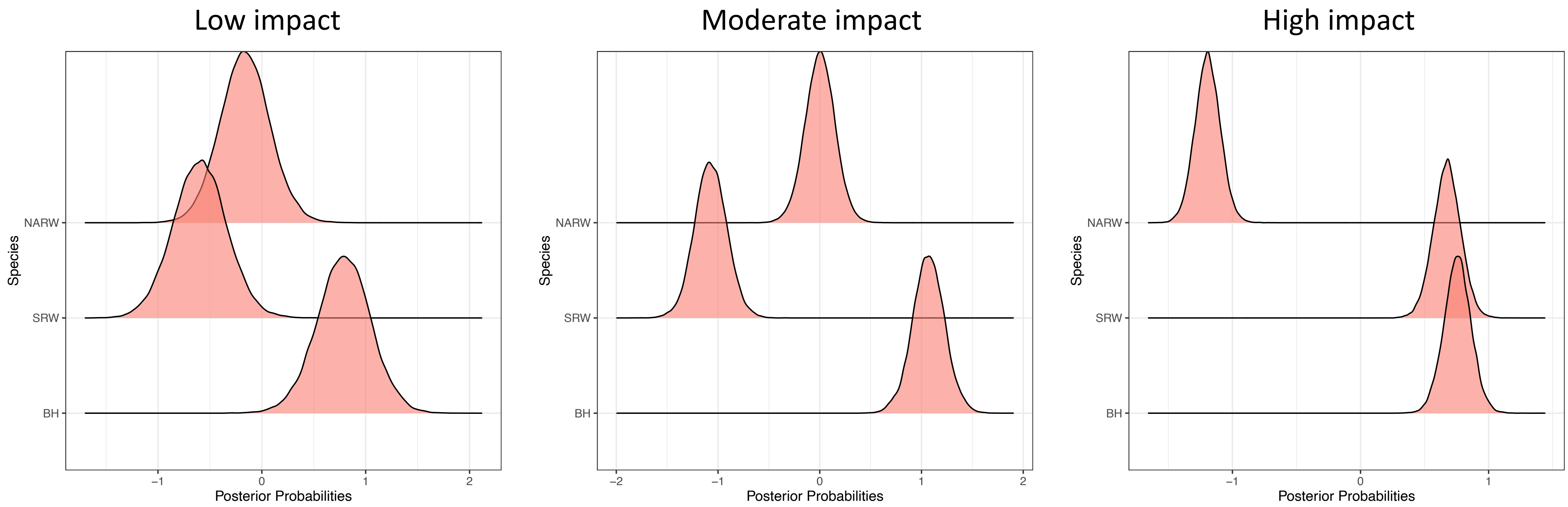

Fig. S11. Posterior probability distributions for the effect of species on derived allele frequencies for low, moderate, and high impact mutations across autosomes.

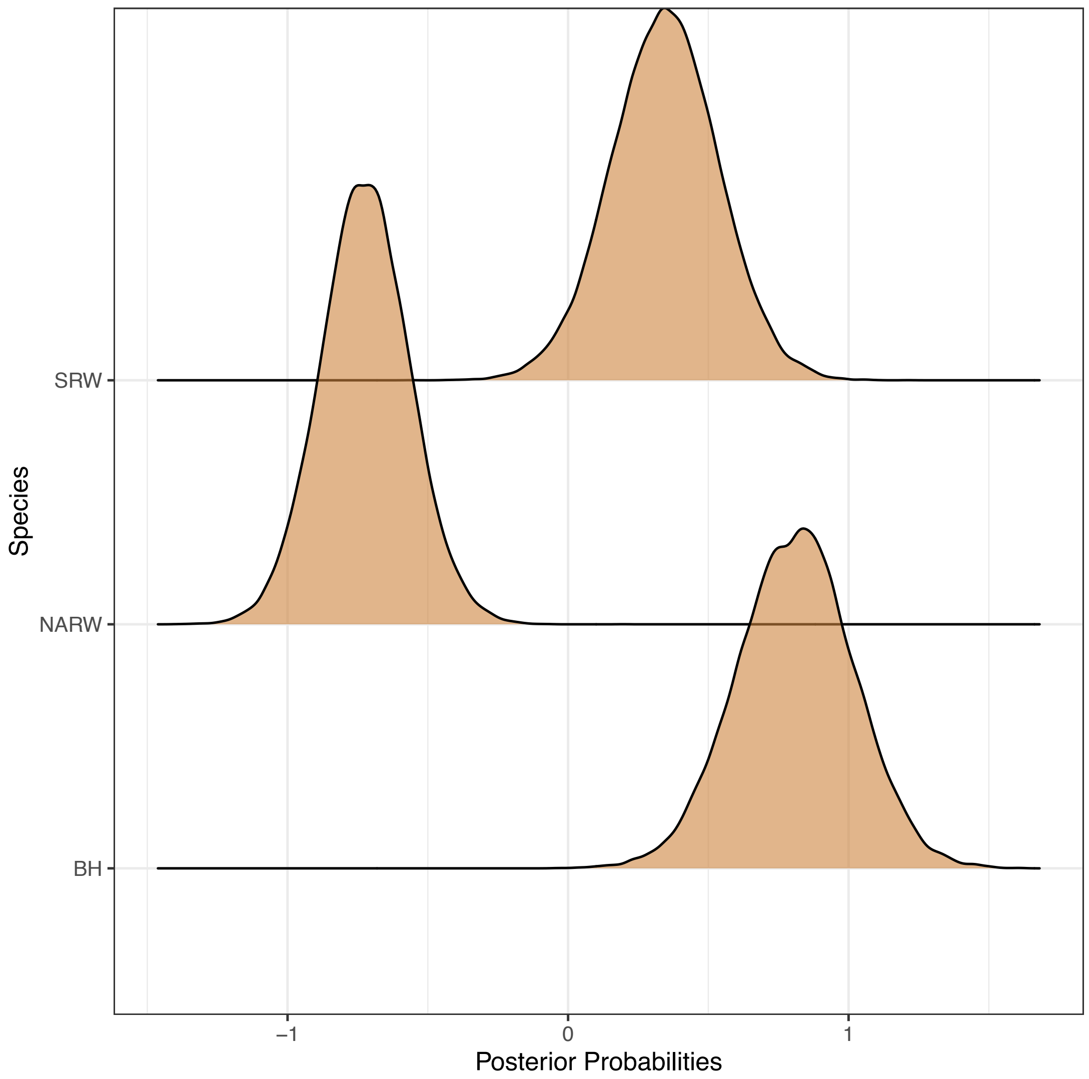

Fig. S12. Posterior probability distributions for the relationship between species and the standardized relative mutation load.

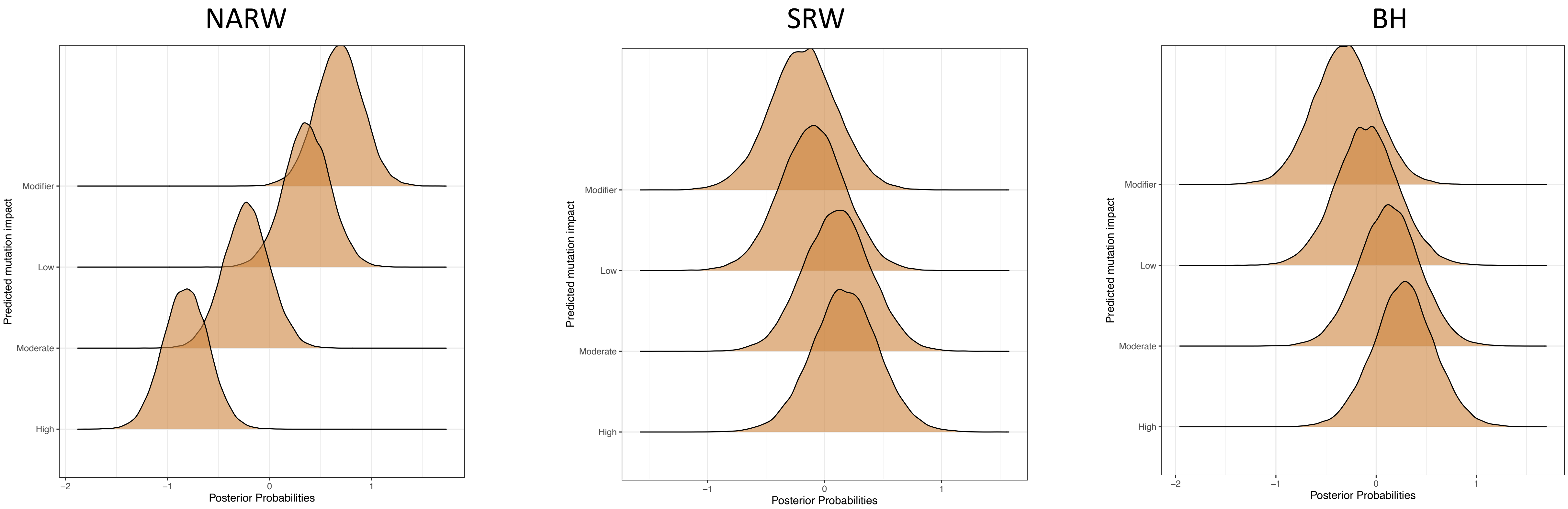

Fig. S13. Posterior probability distributions for the relationship between predicted mutation impact and the standardized enrichment of deleterious variation in runs of homozygosity across autosomes for NARW, SRW, and BH.

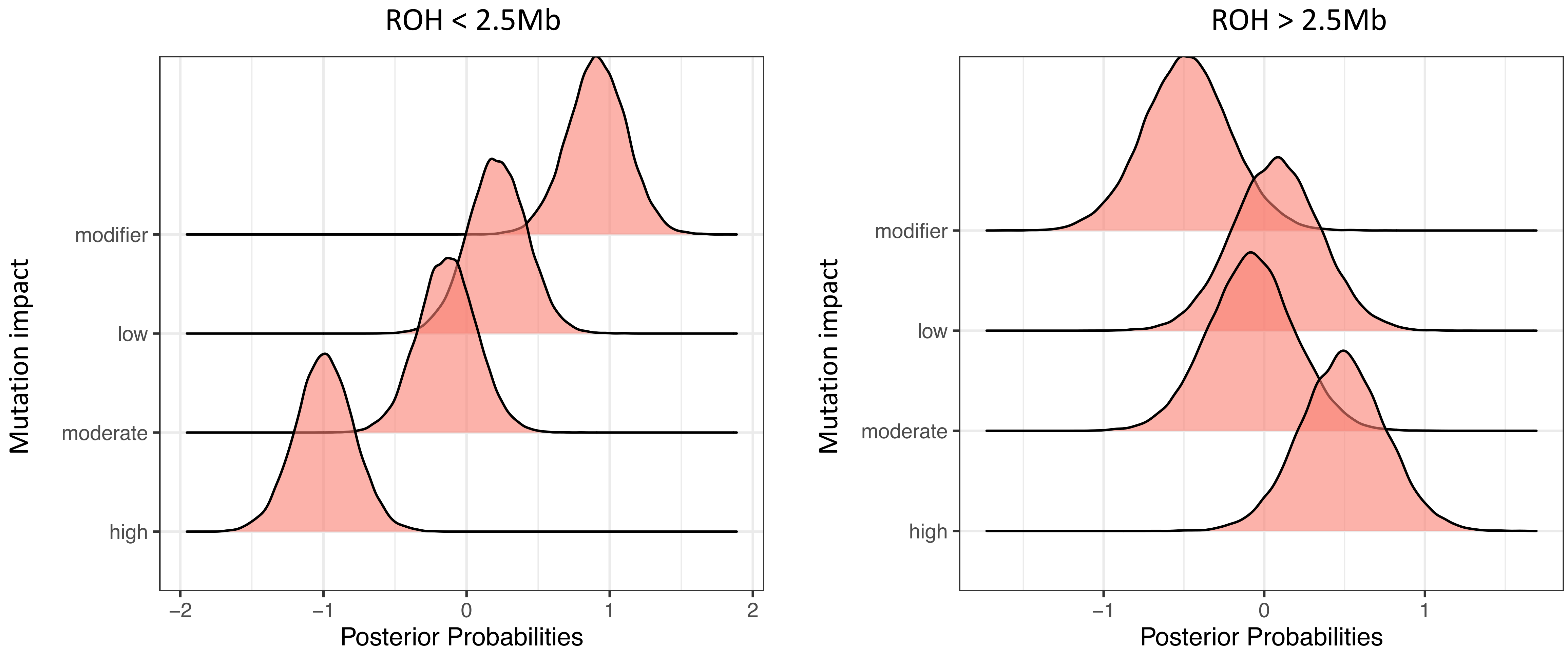

Fig. S14. Posterior probability distributions for the relationship between predicted mutation impact and the standardized enrichment of deleterious variation in runs of homozygosity both less than 2.5Mb and greater than 2.5Mb across autosomes for NARW. Note that ROH > 1Mb are nearly absent across all species within the X chromosome.

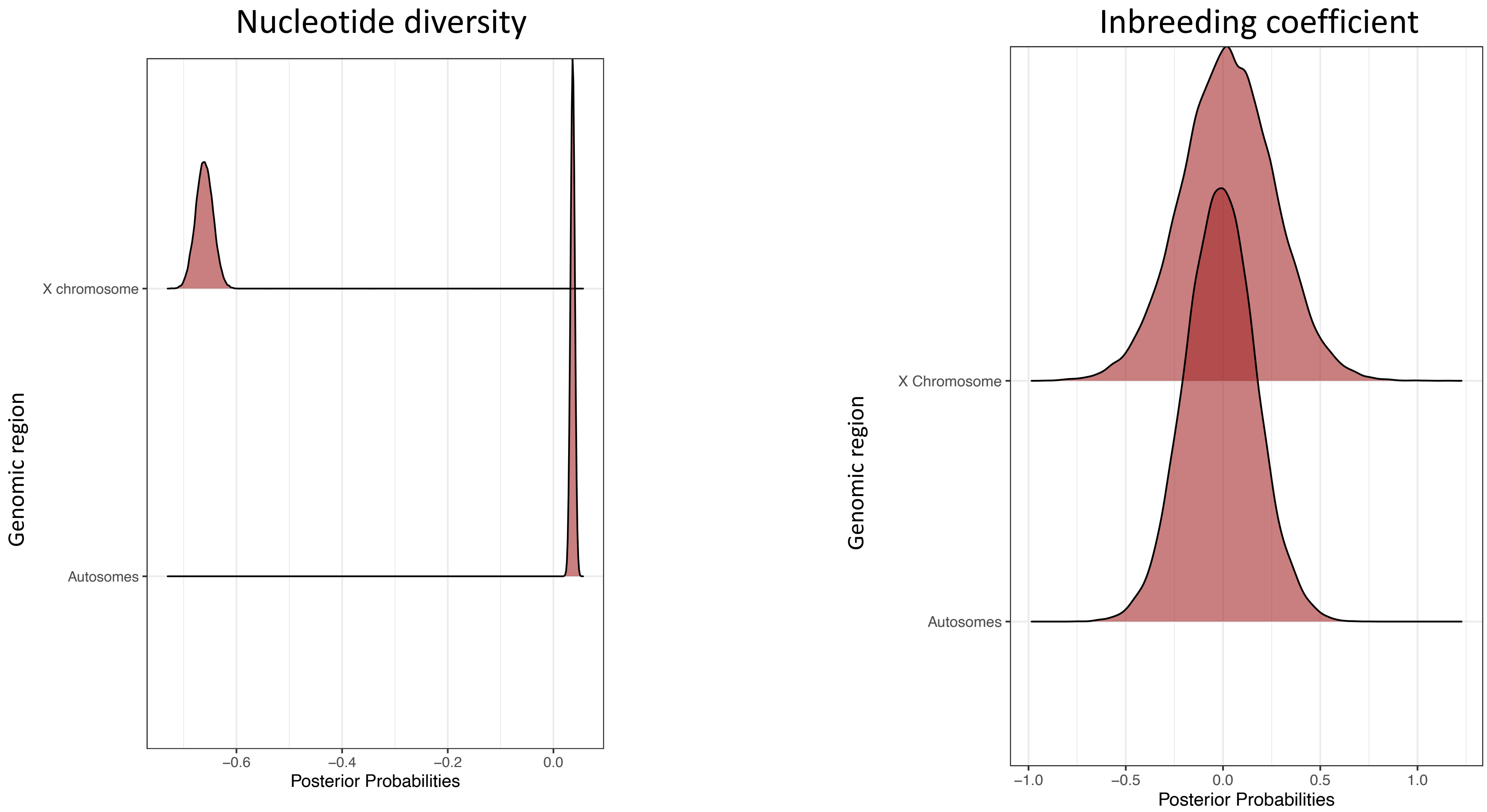

Fig. S15. Posterior probability distributions for the effect of genomic region on standardized nucleotide diversity and the inbreeding coefficient.

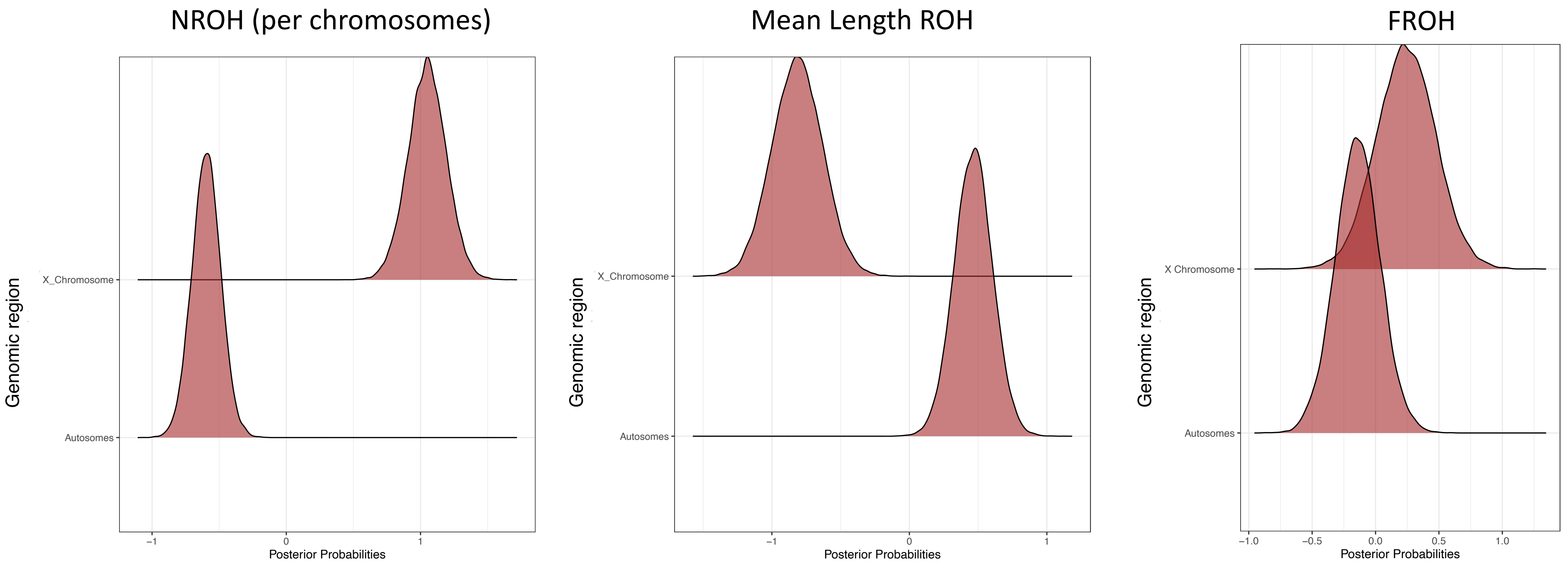

Fig. S16. Posterior probability distributions for the effect of genomic region on the standardized mean number of runs of homozygosity per chromosome, the mean length of runs of homozygosity, and the fraction of the genome captured in runs of homozygosity.

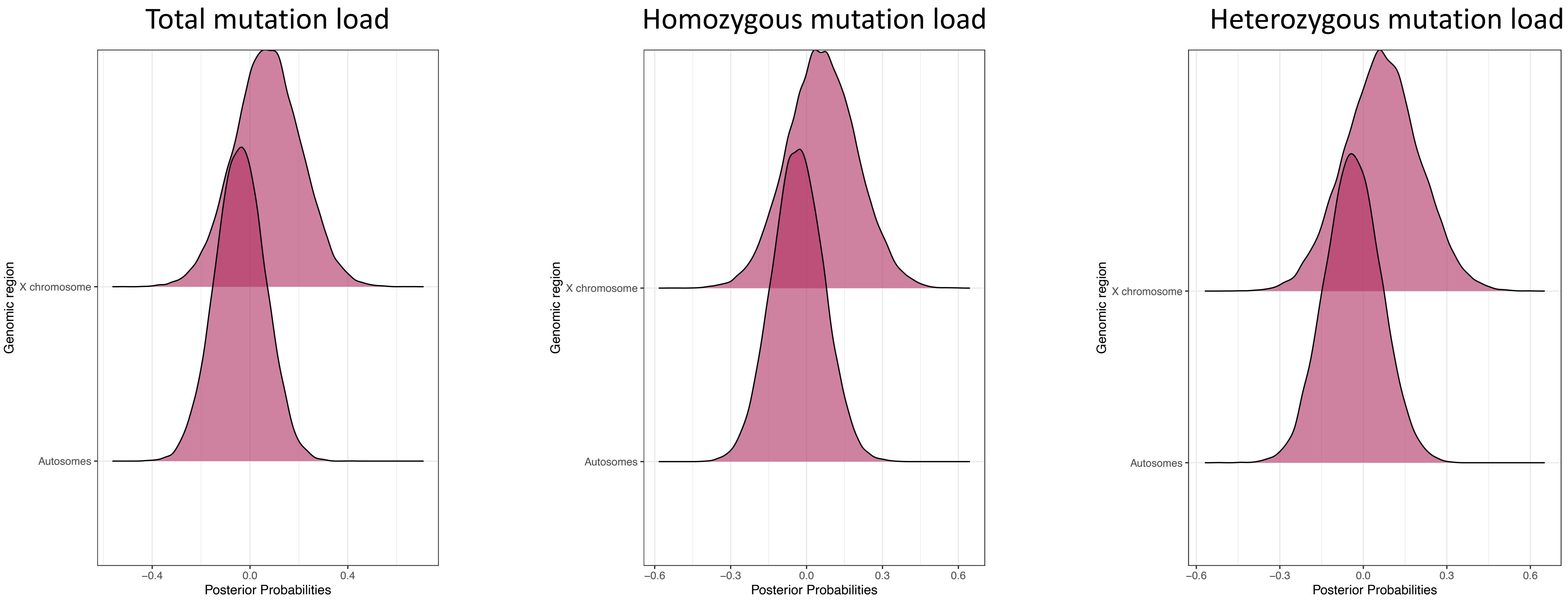

Fig. S17. Posterior probability distributions for the effect of genomic region on the standardized mutation load.

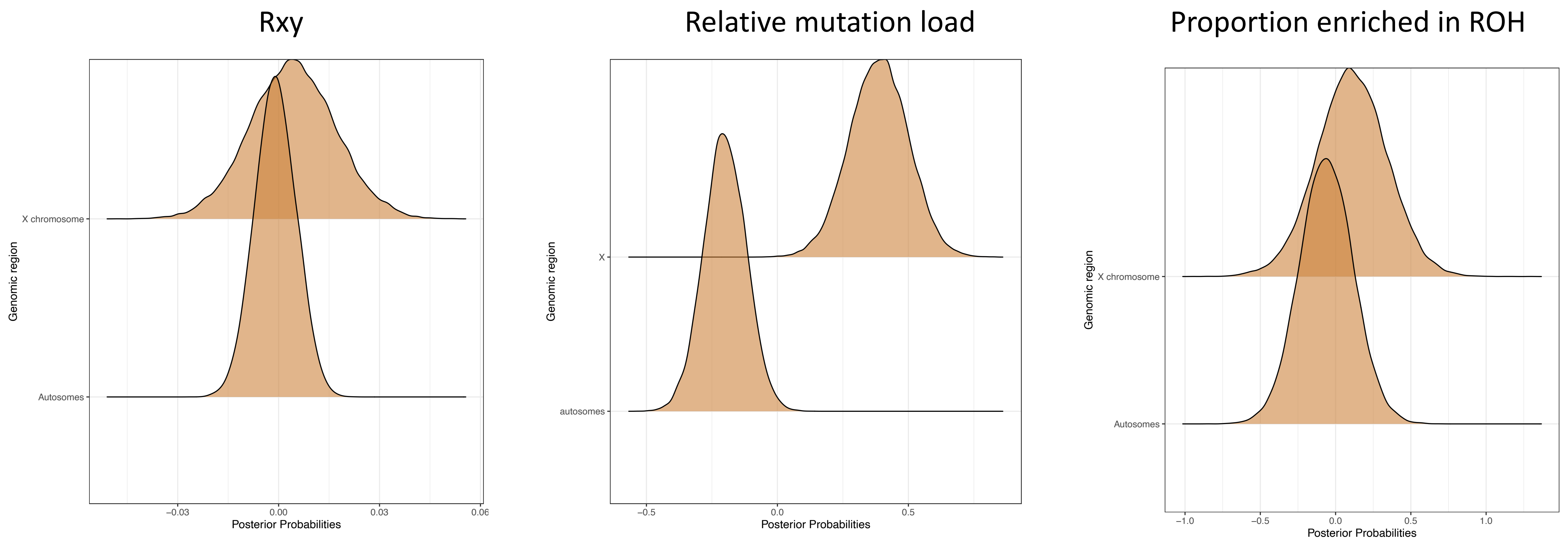

Fig. S18. Posterior probability distributions for the effect of genomic region on standardized *Rxy*, the relative realized mutation load, and the proportion of deleterious alleles enriched in runs of homozygosity.

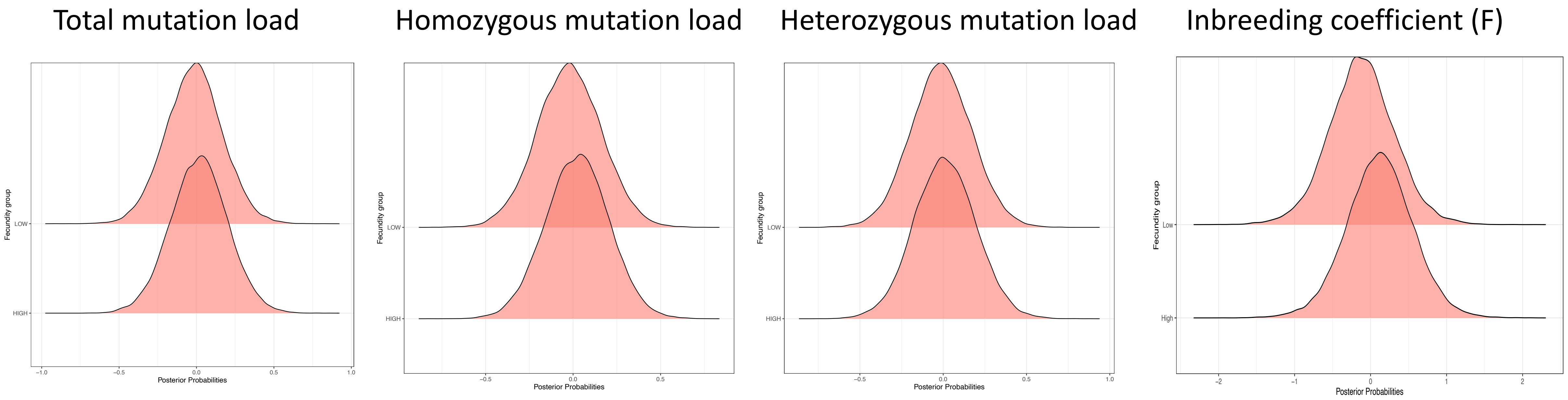

Fig. S19. Posterior probability distributions for the effect of reproductive success on standardized mutation load and inbreeding in NARW.

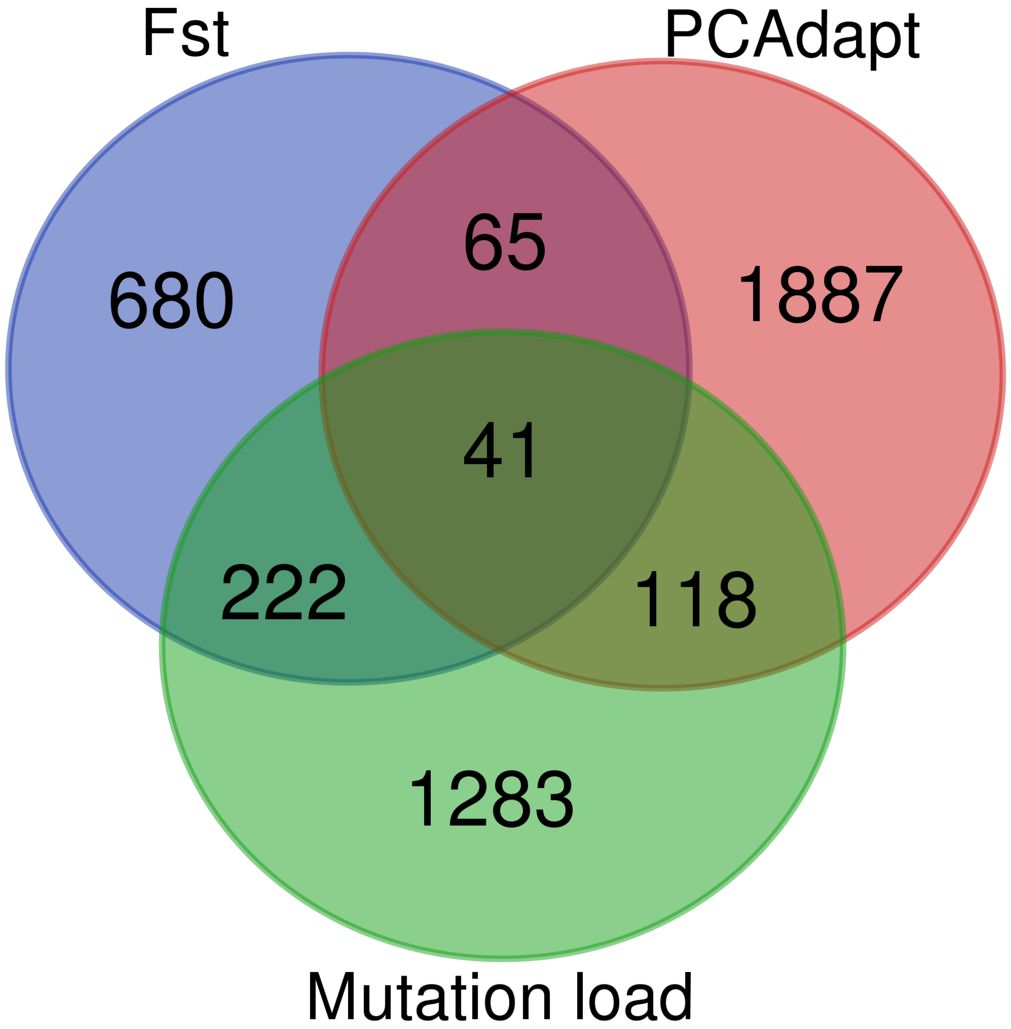

Fig. S20. Venn diagram for the number of candidate variants assigned for each of three outlier approaches based on *Fst*, Principal Components, and mutation load.

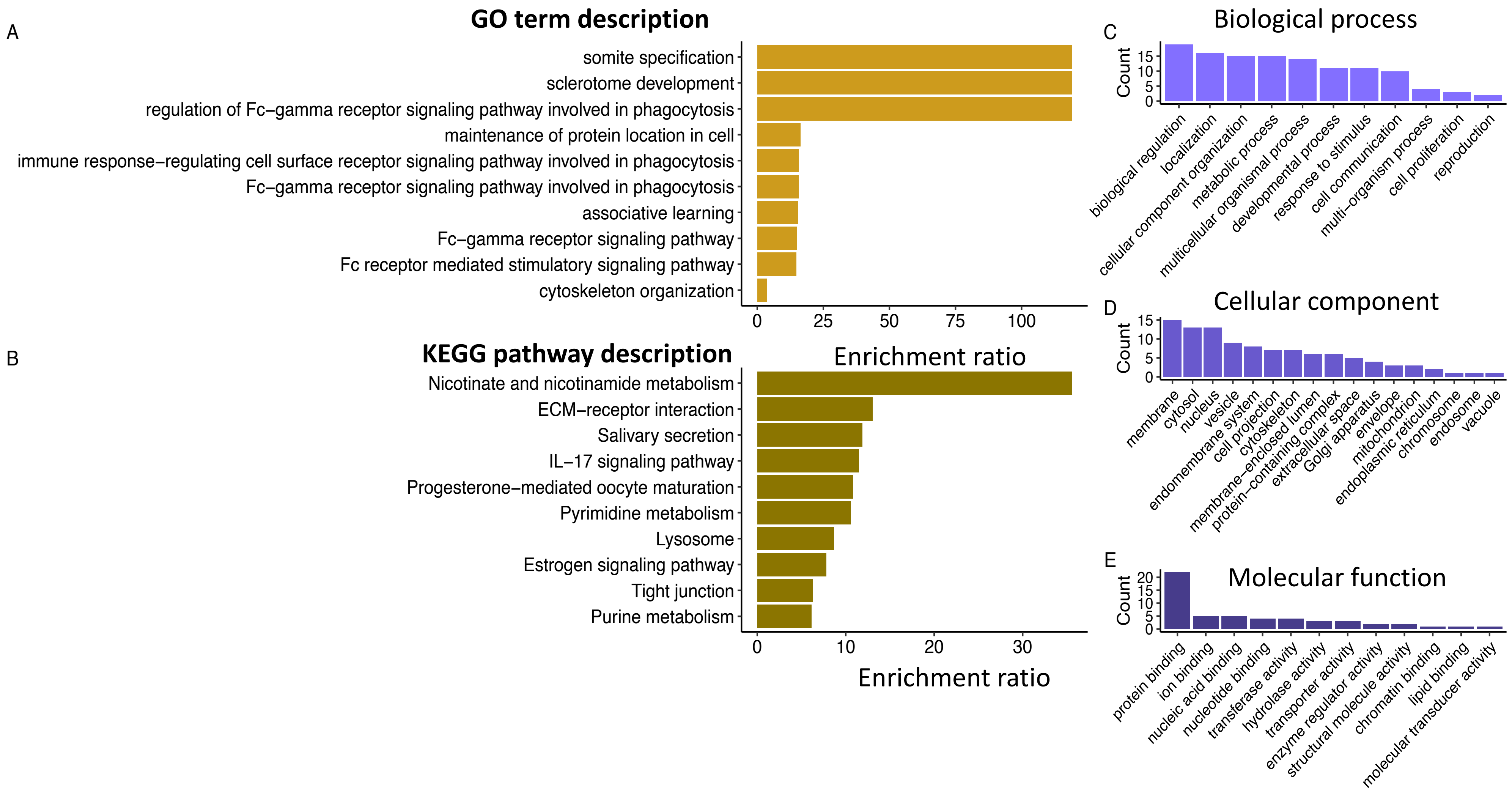

Fig. S21. Full set of results from gene enrichment analyses that includes redundant terms. (A) Terms with the highest gene enrichment values from over-representation analysis using a Gene Ontology background. (B) Terms with the highest gene enrichment values from over-representation analysis using the KEGG background.
