# Supplementary material for: Genomic Evidence for the Purging of Deleterious Genetic Variation in the Endangered North Atlantic Right Whale
Source: Evol Appl. 2024 Dec 23;17(12):e70055. doi: 10.1111/eva.70055 (PMC11665784; doi:10.1111/eva.70055)
Supplement: Supplementary file 2 — Table S1. [file EVA-17-e70055-s001.docx]

Table S1. Number of observed calves for each individual female in the study, with the first and last year sighted. These data come from a 40+ year photo-identification study of the species (77). Note that females with 0 observed calves comprise the “Low RS” fecundity group in this study. The other five females are considered to have “High RS” in this study.

Table S2. Genomic summary statistics for individuals used in this study. Columns designate the individual name, the species name, the genomic region (CHR), the inbreeding coefficient (F), the fraction of the genome capture in RoH (FROH), the total number of RoH (NROH), the sum length of all RoH (SROH), the mean length of RoH (meanLROH), the total mutation load (Total ML), the realized mutation load (Realized ML), and the masked mutation load (Masked ML) for each individual in this study.

Table S3. Candidate variant set with allele frequency per group and gene name annotated. Columns designate the chromosome and the position of each variant in the blue whale reference genome, the allele frequency for each variant in the bowhead (BH), Southern right whale (SRW), North Atlantic right whales with high reproductive success (NARW HRS) and nulliparous North Atlantic right whales (NARW LRS), the SnpEff predicted mutation impact (Predicted im), the NCBI gene abbreviation, and full name of the gene to which each respective variant mapped.

Table S4. The average number of derived alleles per individual for each mutation impact category for each species. Data are shown for the autosomes and X chromosome independently.

|  |  |  |  |  |
| --- | --- | --- | --- | --- |
| **Genomic region** | **Mutation impact** | **BH** | **NARW** | **SRW** |
| Autosomes | High | 68.1 | 64.3 | 67.2 |
| Autosomes | Moderate | 2012.9 | 2102.3 | 1881.6 |
| Autosomes | Low | 2835.4 | 2801 | 2785.2 |
| Autosomes | Modifier | 320020.3 | 311068.7 | 310911.4 |
| X chromosome | High | 16.7 | 13.5 | 11.6 |
| X chromosome | Moderate | 418.7 | 422.8 | 410.2 |
| X chromosome | Low | 578.3 | 500.6 | 514.4 |
| X chromosome | Modifier | 83149.7 | 83670.5 | 82377.2 |
